# Epicuticular wax accumulation and regulation of wax pathway gene expression during bioenergy Sorghum stem development

**DOI:** 10.3389/fpls.2023.1227859

**Published:** 2023-10-23

**Authors:** Robert Chemelewski, Brian A. McKinley, Scott Finlayson, John E. Mullet

**Affiliations:** ^1^ Department of Biochemistry & Biophysics, Texas A&M University, College Station, TX, United States; ^2^ Department of Soil and Crop Sciences, Texas A&M University, College Station, TX, United States

**Keywords:** Sorghum, bioenergy, stem cuticular wax, scanning electron microscopy, wax load, gene regulatory network analysis, policosanol

## Abstract

Bioenergy sorghum is a drought-tolerant high-biomass C4 grass targeted for production on annual cropland marginal for food crops due primarily to abiotic constraints. To better understand the overall contribution of stem wax to bioenergy sorghum’s resilience, the current study characterized sorghum stem cuticular wax loads, composition, morphometrics, wax pathway gene expression and regulation using vegetative phase Wray, R07020, and TX08001 genotypes. Wax loads on sorghum stems (~103-215 µg/cm^2^) were much higher than Arabidopsis stem and leaf wax loads. Wax on developing sorghum stem internodes was enriched in C28/30 primary alcohols (~65%) while stem wax on fully developed stems was enriched in C28/30 aldehydes (~80%). Scanning Electron Microscopy showed minimal wax on internodes prior to the onset of elongation and that wax tubules first appear associated with cork-silica cell complexes when internode cell elongation is complete. Sorghum homologs of genes involved in wax biosynthesis/transport were differentially expressed in the stem epidermis. Expression of many wax pathway genes (i.e., *SbKCS6, SbCER3-1, SbWSD1, SbABCG12, SbABCG11*) is low in immature apical internodes then increases at the onset of stem wax accumulation. *SbCER4* is expressed relatively early in stem development consistent with accumulation of C28/30 primary alcohols on developing apical internodes. High expression of two *SbCER3* homologs in fully elongated internodes is consistent with a role in production of C28/30 aldehydes. Gene regulatory network analysis aided the identification of sorghum homologs of transcription factors that regulate wax biosynthesis (i.e., *SbSHN1, SbWRI1/3, SbMYB94/96/30/60, MYS1*) and other transcription factors that could regulate and specify expression of the wax pathway in epidermal cells during cuticle development.

## Introduction

The cuticle covers the aerial surfaces of most terrestrial plants serving an essential role in the adaptation of plants to the environment. The biogenesis, chemistry, morphology, and function of the plant cuticle has been the focus of extensive research (Jenks et al., 1992; Koch and Ensikat, 2008; Samuels et al., 2008; Bernard and Joubes, 2013; Yeats and Rose, 2013; Lee and Suh, 2015; Busta et al., 2017; Lewandowska et al., 2020; Batsale et al., 2021). The cuticle aids in reducing water loss from plant surfaces, dissipates harmful solar radiation, and helps protect plants from pathogens and insects in part by mediating signaling between plants and beneficial microbes/pathogens (Nwanze et al., 1992; Lewandowska et al., 2020; Cardona et al., 2022). The cuticle is composed of cutin, a polyester comprised primarily of cross-linked C16-C18 fatty acids, intracuticular wax and oils embedded in the cutin layer (Buschhaus and Jetter, 2011), and epicuticular wax located on the external surface of the cutin matrix. Intracuticular and epicuticular waxes are composed of mixtures of hydrophobic aliphatic compounds that include long chain fatty acids, primary and secondary alcohols, esters, aldehydes, ketones and lower amounts of triterpenoids, sterols, and polyketides. Epicuticular wax can form plates, crystals, rods, needles, and tubules on surfaces depending in part on the chemistry of secreted wax (Wettstein-Knowles, 1972; Chambers et al., 1975; Koch and Ensikat, 2008). The composition and thickness of epicuticular wax varies depending on species, organ, surface (adaxial, abaxial), genotype and environmental conditions. The molecular basis of extensive variation in wax composition and load and the functional impact of variation has been the subject of numerous studies.

The genetic and biochemical pathways involved in epicuticular wax formation have been characterized in *Arabidopsis thaliana* aided by an extensive collection of *eceriferum* (*cer*) mutants that have altered cuticle appearance and chemical composition (Bernard and Joubes, 2013; Busta et al., 2017). These studies showed that the main components of wax are derived from C16 and C18 fatty acid thioesters that are synthesized in plastids then hydrolyzed and transported to the endoplasmic reticulum (ER) for conversion to long chain acyl-CoAs by long chain acyl-CoA synthases (LACSs) (Busta et al., 2017). The resulting acyl-CoAs are converted to very-long-chain fatty acids (VLCFAs) with chain lengths of 24-34 carbons by a fatty acid elongase complex consisting of KCS/CER6, KCR, HDC/PAS2, and ECR. Proteins encoded by *CER2, CER26*, and *CER2-like* are involved in elongation that produces C30-C34 acyl-CoAs (Negruk et al., 1996; Pascal et al., 2013; Haslam et al., 2015; Haslam et al., 2017; Xue et al., 2017). The long chain acyl-CoAs (Yeats and Rose, 2013) are directed into alcohol-forming or alkane-forming wax pathways prior to export or are oxidized into a VLCFA. The alcohol-forming pathway, mediated by proteins encoded by *CER4* and *WSD1*, produces primary alcohols and esters, whereas the alkane-forming pathway, involving the gene products of *CER3, CER1*, and *MAH1*, produces aldehydes, alkanes, secondary alcohols, and ketones. The wax components are secreted through the plasmalemma via ABCG-transporters (ABCG12, ABCG11) and carried across the wall space in association with lipid transfer proteins (LTPs) (Bernard and Joubes, 2013; Lewandowska et al., 2020).

The regulation of wax biosynthesis is complex in part because cuticle synthesis and organ growth are coordinated and wax load and composition varies depending on stage of development, organ type, and abiotic and biotic factors (Avato et al., 1984; Medeiros et al., 2017; Bourgault et al., 2020; Lykholat et al., 2020). Transcription factors that regulate wax biosynthesis have been identified through analysis of mutants, association studies, and co-expression analysis. For example, analysis of Arabidopsis wax mutants and biochemical studies led to the identification of MYB94 and MYB96 as direct regulators of *KCS1/2/6, KCR1, CER1/2/*3 and *WSD1* expression (Seo et al., 2011; Lee et al., 2016). Additional R2R3-MYB factors including MYB30 and MYB41 also modulate cuticle and wax formation (Cominelli et al., 2008; Raffaele et al., 2008). Diel regulation of wax biosynthesis in leaves is regulated by MYS1/2, DEWAX, SPL9, CER7 and miR156 (Klein et al., 1996; Hooker et al., 2007; Li et al., 2019; Liu et al., 2022). In addition, the AP2/ERF factors WIN1/SHN1 and WRI1 regulate cutin and wax biosynthesis and in Arabidopsis, WRI1 directly modulates expression of *LACS1, KCR1, PAS2, ECR*, and *WSD1* (Park et al., 2016). Increased wax accumulation in response to water deficit is regulated by induction of *RAP2.4* which encodes an AP2/DREB transcription factor (Yang et al., 2020), and increased expression of *MYB94/96* (Seo et al., 2011).

Sorghum is a genetically diverse drought tolerant C4 grass used for production of grain, forage, and biomass for bioenergy (Rooney et al., 2007; Mullet et al., 2014; Boyles et al., 2019; Tao et al., 2021).The heavy wax loads that accumulate on aerial surfaces of *Sorghum bicolor* have been analyzed to better understand the contribution of wax load/chemistry to sorghum’s exceptional tolerance to drought and heat. Early studies found that higher leaf wax loads on sorghum leaves were correlated with reduced cuticular transpiration (Nobel and Jordan, 1983; Jordan et al., 1984) and that leaf wax load and water use efficiency increased in response to water deficit (Saneoka and Ogata, 1987). Variation in wax loads were also correlated with differences in susceptibility to fungal pathogens (Jenks et al., 1994) and insect-plant interactions (Bernays, 1972; Nwanze et al., 1992). A recent study of the sorghum wax phyllosphere found that *Sphingomonadaceae* and *Rhizobiaceae* families were the major taxa associated with stem wax (Mechan-Llontop et al., 2023). SEM analysis revealed that sorghum leaf sheath wax exhibits a wide range of morphologies including plates, crystals, and tubules (McWhorter and Rex, 1989; McWhorter et al., 1990; Jenks et al., 1992). Analysis of sorghum wax chemistry showed that triterpenoids could have an important impact on cuticular transpiration at high temperatures (Busta et al., 2021). Differences in the wax composition of sorghum leaf blades, leaf sheaths, stems and grain has been documented although the functional significance of these differences requires further investigation (Hwang et al., 2002; Harron et al., 2017; Xiao et al., 2020; Busta et al., 2021; Tang et al., 2022). The availability of variation in wax load among sorghum accessions and the generation and characterization of sorghum wax mutants (Peters et al., 2009; Xin et al., 2009; Xin et al., 2021) has enabled QTL, association, and map-based identification of several sorghum genes involved in cuticle and wax biosynthesis (Burow et al., 2008; Burow et al., 2009; Awika et al., 2017; Punnuri et al., 2017; Uttam et al., 2017; Elango et al., 2020). These studies characterized *WBC11*, an ABC transporter that is involved in wax transport (Mizuno et al., 2013) and a GDSL-lipase homolog of cutin synthase that reduces wax accumulation when mutated (Jiao et al., 2017).

Drought and heat tolerance are especially important traits in bioenergy sorghum because this crop is being developed for biomass production on marginal annual cropland suitable for bioenergy crops. Moreover, bioenergy sorghum’s long vegetative growth duration (>150 days) typically results in exposure to abiotic stress during a significant portion of the growing season. Most of the previous studies of sorghum wax have utilized dwarfed grain sorghum genotypes and focused on wax accumulation on leaf blades and leaf sheaths. In contrast, bioenergy sorghum stems are 4-5 m in length by the end of the growing season and stems, like leaf sheaths, are covered with a heavy wax load. Therefore, the current study focused on characterizing the developmental timing and extent of stem wax accumulation, morphology and composition during plant development and the identification of genes involved in stem wax biosynthesis. This analysis revealed that stem wax loads are very high and that wax composition changes during stem development resulting in the accumulation of high levels of long chain waxy aldehydes on the surface of older stem internodes. Transcriptome and gene regulatory network analysis enabled the identification of transcription factors that are predicted to regulate stem wax biosynthesis.

## Materials and methods

### Plant material

Stem tissues for wax analysis were obtained from TX08001, a photoperiod sensitive late flowering bioenergy sorghum hybrid developed at Texas A&M University, R07020, a photoperiod sensitive bioenergy sorghum inbred, and Wray, a photoperiod sensitive sweet sorghum inbred. Seeds were obtained from the Sorghum Breeding Lab at Texas A&M University in College Station, Texas and planted in the field or in a greenhouse in 6 L pots filled with Jolly Gardner Pro-Line C25 soil or in rhizotrons (20 cm diameter x 75cm long) filled with field soil. TX08001, R07020, and Wray were grown in a greenhouse for 90 days under long day conditions to maintain plants in the vegetative phase. Stem internode tissue was harvested from Wray (74 DAE) and R07020(60 DAE) plants (DAE -Days after Emergence) (Casto et al., 2018; Fu et al., 2023). Stem tissues were harvested from TX08001 at 90 DAE for SEM analysis. TX08001 plants were also grown in rhizotrons for 120 days in an automated phenotyping greenhouse with tall side walls in 2022 and harvested for wax composition analysis. Stem samples were also obtained from field grown TX08001 in 2021 harvested at 125 DAE. Wax was extracted for wax load and composition analysis from TX08001 grown at the Texas A&M University Farm in Burleson County, TX in 2019 and 2020. Field grown plants were fertilized at planting with a solution of liquid ammonium polyphosphate (11-37-0), UAN 32% and zinc sulfate. Field soil is designated Roetex Clay. TX08001 plants were thinned at 21 DAE to 15 cm spacing between plants in rows, with 76 cm spacing between rows. Seeds were treated with Concep III, Nugro, and Apron X. Field plots were not irrigated after planting.

### Scanning electron microscopy analysis

Internode epidermal tissue was excised and placed onto Kimwipes and then put under a low-pressure nitrogen stream for two hours to pre-dry the tissue. The samples were then transferred to a desiccation globe and dried by exposure to low pressure for 48 hours. Samples were attached to 1 cm pin mount stands with Electron Microscopy Sciences conductive double sided carbon tape (8mm). The samples were sputter coated with 7nm of palladium, then analyzed with a FEI Quanta 600 FE-SEM at 10kV under low pressure.

### Stem wax extraction

Plant material (TX08001) was harvested by cutting plants at the base of the stem for deconstruction and wax extraction. Stems were divided into node-internode sections and labeled based on phytomer number (P1 corresponds to the youngest phytomer located at the top of the plant). The surface area of each tissue type targeted for wax extraction was measured while samples were still fresh. Wax extraction was carried out by immersing samples in hexane for 40 seconds followed by the transfer of hexane extracts to preweighed glass autosampler tubes. The sample was then subjected to a second and third hexane extraction of 40 sec each. The three hexane washes were obtained, each was dried to remove hexane, and the weight of extracted wax was summed to calculate wax load. This protocol was developed to optimize the extraction of epicuticular and intracuticular wax while minimizing the extraction of C16 and C18 fatty acids and chlorophyll. The yield of wax decreased and C16/C18 fatty acids increased when more than three 40 sec washes were utilized for extraction. Following three hexane extractions of older stem internodes (>P9) residual wax was still visible on stem surfaces and could be removed with a hexane-soaked cloth. This additional wax fraction was not included in the wax yields presented in this paper.

### Wax load calculation

TX08001 internode diameters were measured with calipers to a tenth of a millimeter above the pulvinus (Base of the Internode), below the nodal plexus (Top of Internode) and at the mid-point of the internode (Middle of the Internode) which typically has the smallest diameter. The length (h) of the internode was also measured with calipers to a tenth of a millimeter. Surface area was then estimated based on a model of stem internodes as the walls of two intersecting truncated cones where the largest diameter of each cone corresponded to the ends of the internode (resulting in two trapezoidal calculations). $Surface\;Area=\left(\frac{h}{2}\right)\left(Top\;of\;Internode+Middle\;of\;Internode\right)\pi /2$ + (h/2)(Middle of internode+Base of Internode) π/2

### GC-MS analysis

TX08001 waxes were transferred in hexane to autosampler vials for analysis in 500 µg aliquots, then dried under a nitrogen stream before adding 100 uL of BSTFA (with 1% TCMS) and incubating at 85°C for 12 hours. Excess BSTFA was evaporated under nitrogen and chloroform was used to suspend the sample at 500 µg/mL. Samples were then analyzed on an Agilent 7890A/7693A/5975C XL GC-MS equipped with a 30-m DB-5MS column (0.25-mm film) using pulsed splitless injection (290°C inlet). Helium was used as the carrier gas at 105.75 mL/min. The oven was held at 70°C for 1 min, then ramped at 10°C/min to 200°C, then ramped at 4°C/min to 295°C and maintained at 295°C for 20 min (total run time 57.75 minutes). The ion source was maintained at 230°C and the quadrupole at 150°C. Mass charge selection for the quadrupole was set to 198-552 m/z (Yang et al., 2017).

High temperature GC-MS was performed by the Thermofisher Demo Lab on a Thermo Scientific ISQ 7000. Bulk wax was resuspended in hexane and transferred to autosampler vials in 1 mg aliquots, then dried under a nitrogen stream. Samples were resuspended in chloroform at a concentration of 1mg/mL and separated on a RESTEK Rxi-5HG column (15m x.25mm x.1µm). The inlet and detector were set to 390°C, and the oven was set to ramp from 120°C to 240°C at a rate of a of 15°C/min and then from 240°C-390°C at 8°C/min and maintained at 390°C for 6 minutes. Samples were injected at a volume of 1µL at concentrations ranging from 400-2000µg/mL (in chloroform). Mass charge selection for the quadrupole was set to 48-952 m/z (Tada et al., 2014).

### RNA sequencing

Three replicates of stem plant tissues were harvested for RNAseq analysis. Some of the R07020 stem samples were cored to separate rind and stem core tissues prior to RNA extraction. The stem tissue samples were placed in 50 mL conical tubes, frozen in liquid nitrogen and stored at -80 C. The tissue was then ground into a fine powder using a mortar and pestle cooled with liquid nitrogen before RNA was extracted using the Zymo Research Direct-zol RNA MiniPrep Plus kit. RNA quality was checked using an Aglient Bioanalyzer then sent to the Joint Genome Institute for library construction and sequencing at a read depth of 30-50 M reads/replicate. Sequenced reads were aligned to the *Sorghum bicolor* V3.1 genome using HISAT2 aligner. The transcriptome assembly and TPM normalization were conducted using String Tie version 1.3 and relative expression used TPM normalized data. RNAseq data from two prior studies was used in the current study (Casto et al., 2018; Fu et al., 2023). Four replicates of Wray stem internode tissue were harvested from vegetative stage plants at 74 DAE, frozen, sent to the Pacific Northwest National Laboratory where stem cell types were isolated using laser capture microdissection (LCM) and RNAseq analysis was done to identify cell-type transcriptomes. The details of LCM methodology, RNA extraction and library construction are described in (Fu et al., 2023). RNAseq data derived from LCM analysis was processed as described above and used to examine the expression of wax pathway genes in stem cell types and for gene regulatory network analysis.

### Phylogenetic analysis assisted identification of sorghum wax pathway genes

Sorghum genes involved in stem wax biosynthesis were identified in a multi-step process. Sorghum homologs of validated wax pathway genes characterized in *Arabidopsis thaliana*, *Oyrza sativa*, and *Zea mays* were identified initially through Blast X analysis. Sorghum homologs with an E-value of 1e^-20^ or lower that encoded proteins with the same PFAM domains as the protein encoded by a validated target wax pathway gene were used for phylogenetic analysis using MEGA X (Tamura et al., 2011; Kumar et al., 2018; Tamura et al., 2021). Alignments were performed with Clustal-W and were co-validated with MUSCLE. The phylogenetic tree was constructed using maximum likelihood with the Jones-Taylor-Thornton amino acid substitution model and all families were bootstrapped with at least 500 replicates. The MYB phylogenetic tree was produced as described in (Singh et al., 2020). The phylogenetic analysis conducted in the current study extended a prior analysis of sorghum MYB factors carried out by (Singh et al., 2020) by including SbMYB86, a gene is relevant to this study. During review, it was noted that the results of our sorghum MYB phylogenetic analysis were consistent with a prior multi-species phylogenetic analysis of MYB factors (Hennet et al., 2020). Sorghum genes that clustered with the validated wax pathway gene at nodes with >80% bootstrap support (*SbLACS9* and *SbWSD1* failed this threshold **Supplemental Figure 3A**) were analyzed further to identify genes that were differentially expressed in the stem epidermis of internode 4 (Wray) compared to other stem cell types (pith parenchyma, xylem, phloem, vascular parenchyma) (Fu et al., 2023). The subset of sorghum wax pathway genes that were differentially expressed in the epidermis were used for wax pathway gene regulatory network (GRN) analysis and to investigate the expression of these genes during bioenergy sorghum internode development.

### Gene regulatory network analysis

Stem cell type transcriptome data obtained by laser capture microdissection (LCM) (Fu et al., 2023) was used to construct a wax pathway gene regulatory network (GRN). The general approach and methods used for gene regulatory analysis have been previously described (Varala et al., 2018; Yu et al., 2022; Fu et al., 2023). Genes involved in wax biosynthesis are differentially expressed at higher levels in the stem epidermis compared to other stem cell types. Transcriptome profiles of stem epidermal cells, pith cells, vascular bundle sclerenchyma, phloem, and vascular bundle parenchyma were obtained in a separate study using laser-capture microdissection and RNAseq analysis (Fu et al., 2023). The stem cell type transcriptome profiles were used to identify genes encoding transcription factors that were differentially expressed in epidermal cells (vs. the other stem cell types), that are co-expressed with wax pathway genes, and that have predicted binding sites in the promoters of the wax pathway genes and TFs that are part of the wax pathway gene regulatory network. To do this, data from each cell type replicate was summed across transcript-level TPMs and counts to obtain gene-level TPMs and counts. Next, an expression threshold was applied to retain genes that exhibited expression of TPM ≥ 5 in at least one sample (one sample being the mean of three biological replicates). TPM normalized expression was used instead of count data for expression thresholding because TPM values allow for comparison across samples and genes. Next, read counts for the same gene set were used to calculate differential expression (DE) with the edgeR package. To calculate DE, gene-level read counts were normalized using Trimmed Mean of M-values normalization. DE is calculated using two groups. The first group was comprised of the epidermis of the LCM dataset. The second group was comprised of bundle sheath, phloem, pith parenchyma, xylem/vascular parenchyma, and xylem cell types of the LCM dataset. We contrasted the epidermal cells relative to the other cell types and defined the genes that were positively or negatively differentially expressed in the epidermis as upregulated or down-regulated, respectively. Following DE analysis, the dataset was further constrained to genes that exhibited DE ≥ 5, FDR < 0.05 (in addition to being expressed > 5 TPM in one triplicate sample). The TPM data of the genes that were DE were used to construct the GRN by integrating three different metrics: (i) Pearson’s correlation coefficient (PCC); (ii) Mutual Rank (calculated as the geometric mean of the ranking of each gene in the PCC rank of the other gene of the pair); and (iii) the capacity of one of the genes of the pair to bind to the promoter of the other gene (i.e., one of the genes being a TF and the promoter of the other gene possessing an enriched conserved regulatory element (CRE) that could be bound by the TF). To facilitate the third criterion, the putative promoter sequences of all genes in the sorghum genome were subjected to DNA pattern analysis using the position weight matrix available in Plant Promoter Analysis Navigator–PLANTPAN3.0; (http://plantpan.itps.ncku.edu.tw/index.html) (Chow et al., 2019) to match known CREs with sequences in sorghum promoters. Promoter sequences annotated spanned 1 kb upstream of the transcription start site. Transcription start site locations were obtained from the Morokoshi sorghum transcriptome database (Makita et al., 2015). As construction of the network considers the potential for promoter binding as described above, every edge in the network consists of at least one TF. The GRN was constructed by selecting all the edges that had PCC ≥ 0.9, FDR ≤ 0.05 and where at least one of the two genes was a TF capable of binding to an enriched CRE in the promoter of the other gene in the pair. When a PCC of -0.9 was used to analyze down-regulated genes, no down-regulated TFs were part of the GRN. If a PCC of -0.7 was used, the down-regulated TFs incorporated into the GRN did not have direct connections to wax pathway genes. Therefore, GRN analysis focused only on genes that were upregulated in stem epidermal cells. The GRN that included genes >5-fold upregulated in stem epidermal cells consisted of 626 genes, and 1416 edges. These statistical thresholds were selected because they resulted in a GRN that included genes that, based on previous knowledge, are likely involved in wax biosynthesis. Further analysis and rendering of the network were conducted in Cytoscape. The GRN network file, the script used to calculate the GRN, and the Cytoscape session file are included in the **Supplemental Materials** and are hosted at https://github.com/brianamckinley/Chemelewski_Wax_2023. In depth analysis of sorghum stem cell type specific gene regulatory networks is described in (Fu et al., 2023).

## Results

### SEM analysis of epicuticular wax deposition during stem development

During sorghum’s adult vegetative phase, a new phytomer (P) is formed immediately below the shoot apical meristem approximately every 3-4 days in good growing conditions. During phytomer development, a leaf blade-sheath grows out from the stem nodal plexus, followed by elongation of the stem internode below the nodal plexus. Stem tissue associated with the youngest 3-4 apical phytomers (all following phytomer (P) numbers are assuming 3 apical phytomers) are short, contain minimal internode tissue, and increase in size primarily through cell division (Kebrom et al., 2017; Yu et al., 2022) (**Supplementary Figure 1**). The onset of stem internode growth is observed in P4 and continues for 9-12 days through the generation of additional cells by the intercalary meristem located at the base of the internode and the elongation of cells in a region immediately above the intercalary meristem (Yu et al., 2022) (**Supplementary Figure 1**). Cessation of internode cell elongation is followed by secondary cell wall formation in a zone of maturation at the upper end of a developing internode. The zone of maturation increases in length during internode growth, eventually spanning the entire internode once cell division ceases (**Supplementary Figure 1**). In a prior study (Kebrom et al., 2017; Yu et al., 2022), the first short internode that became visible below the shoot apex was derived from P4 and was designated Internode #1 (Int#1); internodes at later stages of development were numbered sequentially (Int#2, P5; Int#3, P6, etc.). In the current study, visual inspection of the external surface of stem tissue associated with P1-P4 showed minimal wax accumulation, however, by P7 (Int#4), a white wax layer was visible covering the majority of the internode.

**Figure 1:**
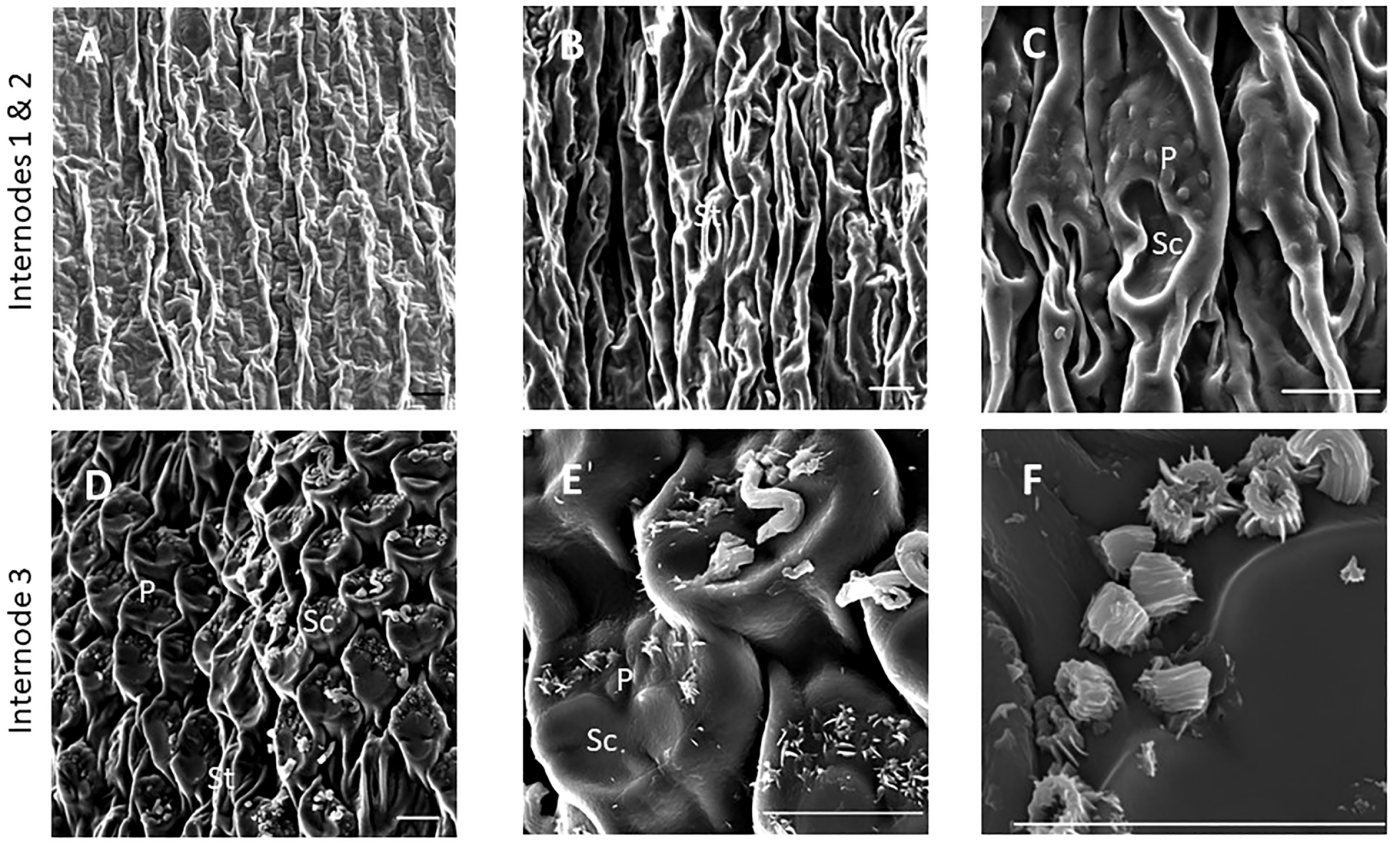
SEM micrographs of the epidermis of TX08001 stem internodes at various stages of development (scale bar = 10 μm). **(A)** Internode 1, the youngest internode analyzed shows minimal wax accumulation. **(B, C)** Stem epidermal tissue taken from the upper end of internode 2 showing stomata [St], silica cells [Sc], and papillae [P]. **(D–F)** Stem epidermal tissue taken from the upper end of the internode 3 showing the onset of wax tubule formation associated with silica cells and papillae. A diagram showing the location of SEM stem sample collection is provided in **Supplementary Figure 1**.

**Figure 2:**
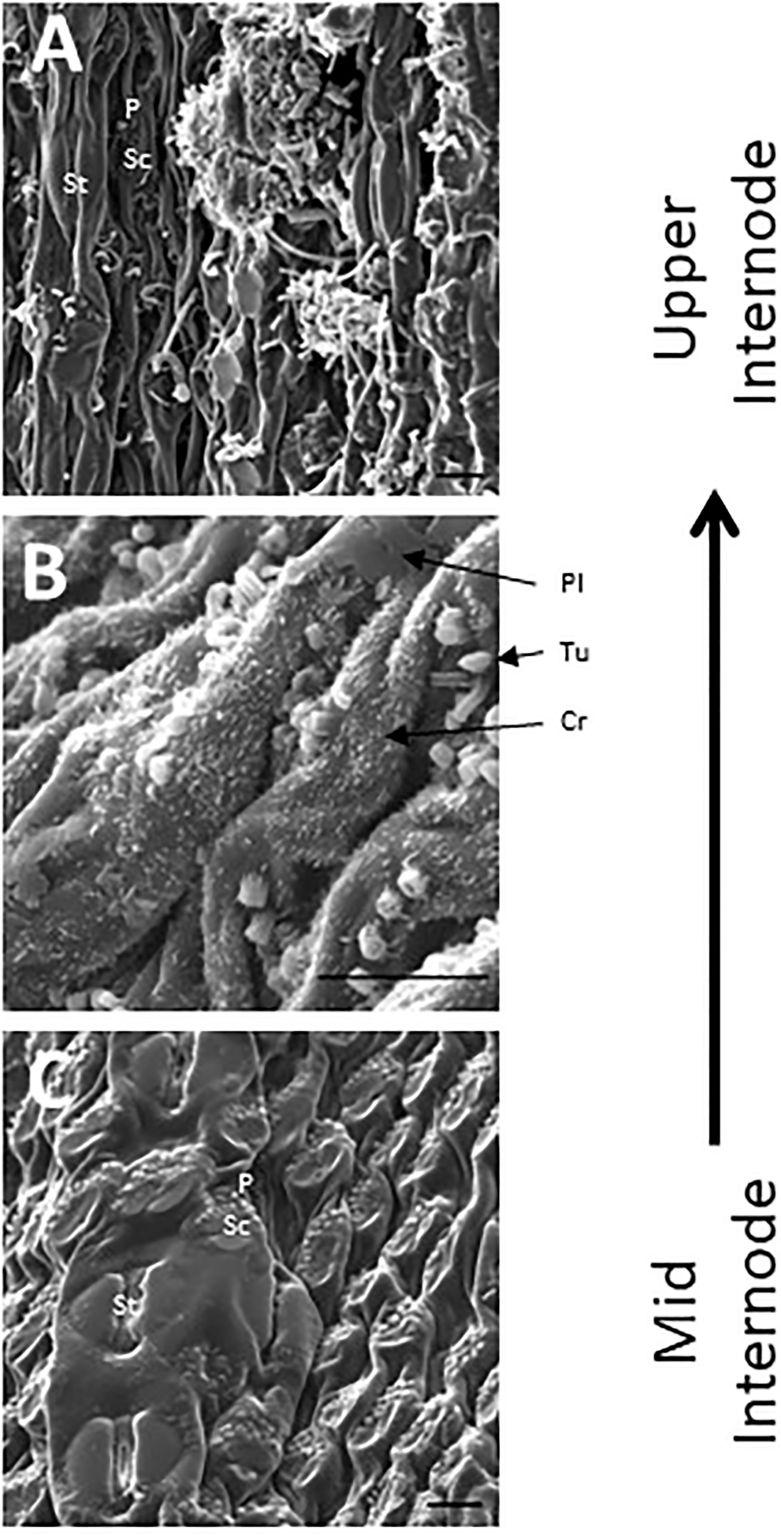
SEM micrographs of the stem epidermis of TX08001 across the upper portion of internode 4 (scale bar = 10 μm). **(A)** SEM of the upper end of internode 4 showing long wax tubules, globular and wax crystals accumulating on epidermal tissue. **(B)** Accumulation of short wax tubules and wax crystals on the surface of less developed internode tissue. Plate wax (Pl), Wax Crystals (Cr), Tubular Wax (Tu). **(C)** SEM aided visualization of silica cells and papillae and minimal wax accumulation on stem tissue taken from the middle of internode 4. A diagram showing the location of SEM stem sample collection across internode 4 is provided in **Supplementary Figure 1**.

**Figure 3:**
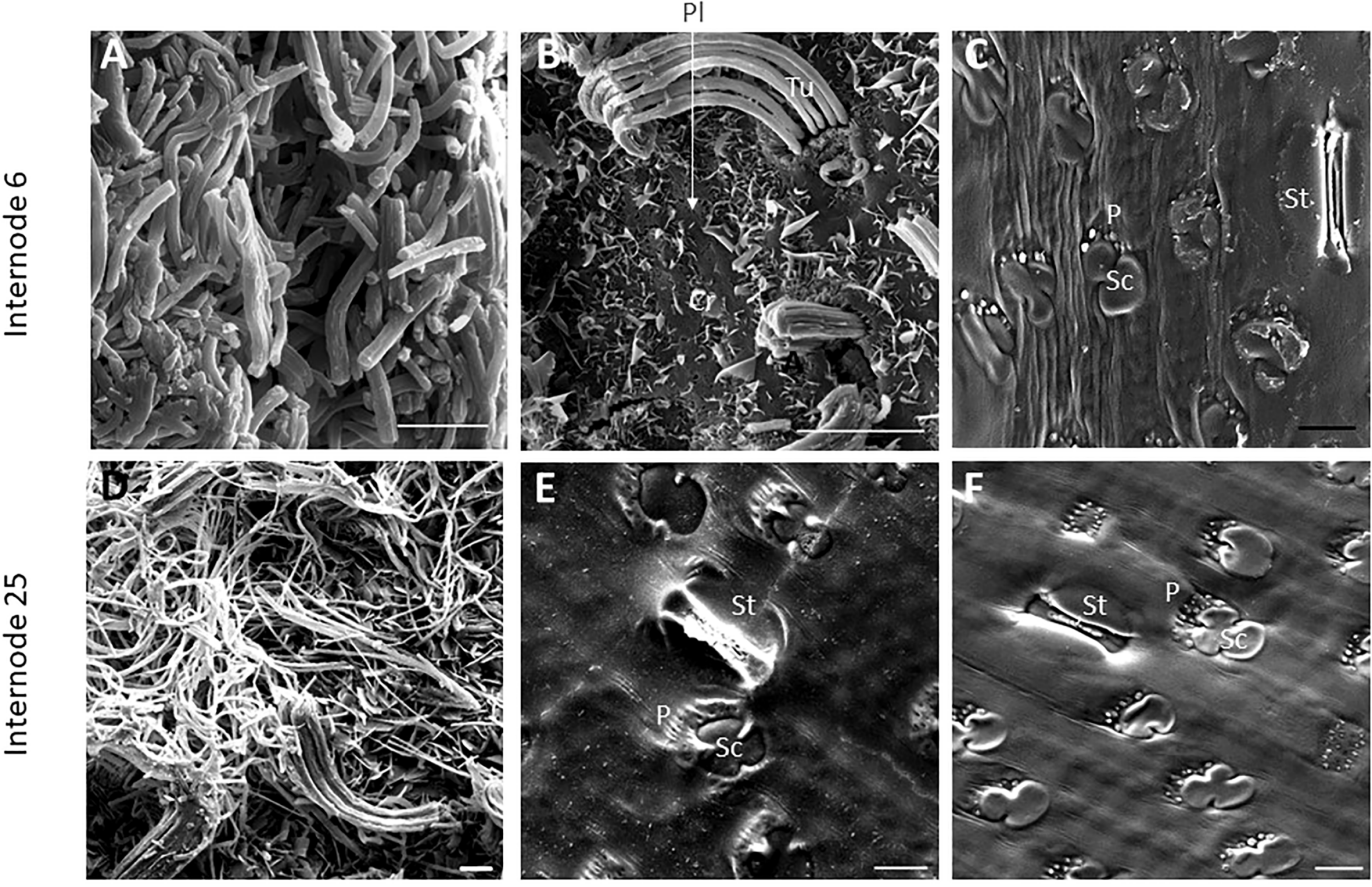
SEM micrographs of fully elongated TX08001 internodes (scale bar= 10μm). **(A, B)** SEM images of Internode 6 showing dense mats of wax tubules **(A)** and regions with fewer wax tubules that appear to be secreted in rows through the cutin layer **(B)**. **(B)** Plate wax (Pl), Wax Crystals (Cr), Tubular Wax (Tu). **(C)** SEM images of a hexane washed section of internode 6 showing epidermal cells including stomata (St), silica cells (Sc) and papillae (P) (image **Supplementary Figure 7** is the non-contrast adjusted image). SEM images of the epidermis of internode 25 **(D–F)** showing wax tubules, filaments, and scales. **(E)** SEM image of the underside of the wax plate on internode 25 showing a ‘negative’ impression of the surface of the differentiated structures. **(F)** The cuticular and epidermal surface of internode 25. To evaluate if the same patterns of secretion were occurring, the wax was peeled to view the back side of the wax mat showing that it coats the surface of the cutin.

SEM has been used to visualize wax accumulation and morphology on sorghum surfaces (McWhorter and Rex, 1989; Jenks et al., 1992). In the current study, SEM was used to examine the deposition of wax on the external surface of stem internodes during internode development (**Figures 1**–**3**). Consistent with visual inspection, SEM analysis showed minimal wax on Int#1 (P4) and Int#2 (P5) (**Figure 1**, Int#1 & 2, A-C) (**Supplementary Figure 1**, locations of SEM sampling). However, stomata (St), putative silica cells (Sc) and nascent papillae (P) that later are associated with wax extrusion were observed on the epidermis of Int#2 (**Figure 1C**). The apical stem tissues are soft and following SEM sample preparation the external cell layer of stem Int#1 (P4) and Int#2 (P5) showed a range of contours and folds. Epicuticular wax was visible at the apical end of Int#3 (P6) distal to the zone of cell elongation (**Figures 1D–F**). The short wax tubules observed on the upper end of Int#3 were associated with the apical end of cells shaped like a clover leaf that have been previously identified as silica cells (Sc) (**Figures 1D–F**) (Jenks et al., 1992). The wax forming on these cells had a tube-like structure (wax tubules) and was associated with papillae (P) (**Figures 1E, F**). Multiple papillae were almost always located at one end of the silica cells (Jenks et al., 1992). SEM analysis of wax accumulation at the middle and upper end of Int#4 is shown in **Figure 2**. Minimal wax was observed on epidermal tissue from the middle of Int#4 (**Figure 2C**), a region of the internode just above the growing zone. This is contrasted by the apical end of the internode spanning fully elongated cells that were covered with epicuticular wax (**Figure 2A**). The morphology of wax that accumulates on fully expanded internodes of older phytomers was also analyzed (**Figure 3**, Int#6, Int#25). Portions of Int#6 (P9) were covered with long wax tubes (**Figure 3A**) which appeared to be extruded through the cutin layer possibly from rows of papillae (**Figure 3C**). Wax crystals and plate-like wax was also observed on the surface (**Figure 3B**). Hexane removal of wax from the surface of Int#6 revealed cloverleaf shaped silica cells (Sc) and associated papillae (P) (**Figure 3C**). SEM analysis of Int#25 from an older plant showed the presence of a dense mat of wax tubes of different diameters (**Figure 3D**). Removal of the wax plate through mechanical lifting revealed a near perfect cast of the underlying epidermal cells (**Figure 3E**) and clover leaf shaped silica cells and associated papillae (**Figures 3E, F**). On occasion, papillae unassociated with a silica cell were observed on the external surface (**Figure 3F**).

### Wax load and GC-MS analysis of wax composition during stem development

Wax extraction from bioenergy sorghum stems was optimized to maximize recovery of wax components while minimizing extraction of C16 and C18 fatty acids and chlorophyll (see methods). Previous estimates of wax loads on grain sorghum leaf sheaths ranged from ~38-206 ug/cm^2^ (McWhorter et al., 1990; Burow et al., 2008; Xiao et al., 2020). In the current study, the wax load from a pioneering survey in 2019 on internode #8 of field grown TX08001 at 90 DAE was ~200 ug/cm^2^ and the wax load on leaf sheath #8 was ~42 ug/cm^2^. In 2020, the wax load on internode #8 of field grown TX08001 plants at 60 DAE was ~103 ug/cm^2^ and the leaf sheath wax load was ~51 ug/cm^2^. Internode #8 (60 DAE) became internode #23 at 120 DAE due to the production of additional phytomers by the shoot apical meristem. By 120 DAE, the wax load on internode #23 was ~215 ug/cm^2^ (**Table 1**). High variance in wax load estimates from internode #23 could be due to loss of wax flakes during plant collection/deconstruction prior to wax extraction and the wax flaking into solution during extraction. However, when the removal of residual wax with a cloth was factored in (see Stem Wax Extraction in Methods), the variance was greatly reduced suggesting this is likely variance associated with the hexane-based wax extraction method.

**Table 1:** Wax load on field grown TX08001 stem and leaf sheaths.

Sample	Time Point	Wax Load (µg/cm^2^)
2019		
Sheath 8	90 DAE	41.2 (3.8)
Internode 8	90 DAE	197 (10.7)
2020		
Sheath 8	60 DAE	51.1 (26.6)
Internode 8	60 DAE	103 (7.3)
Internode 23	120 DAE	215 (117)

Stem internode and leaf sheath samples were collected from phytomer 8 (2019, 2020) and phytomer 23 (2020) of field grown plants and wax load was determined by hexane removal of wax from plant surfaces. The dry weight of wax is reported as µg/cm^2^ of organ surface area (Standard deviation).

**Figure 4:**
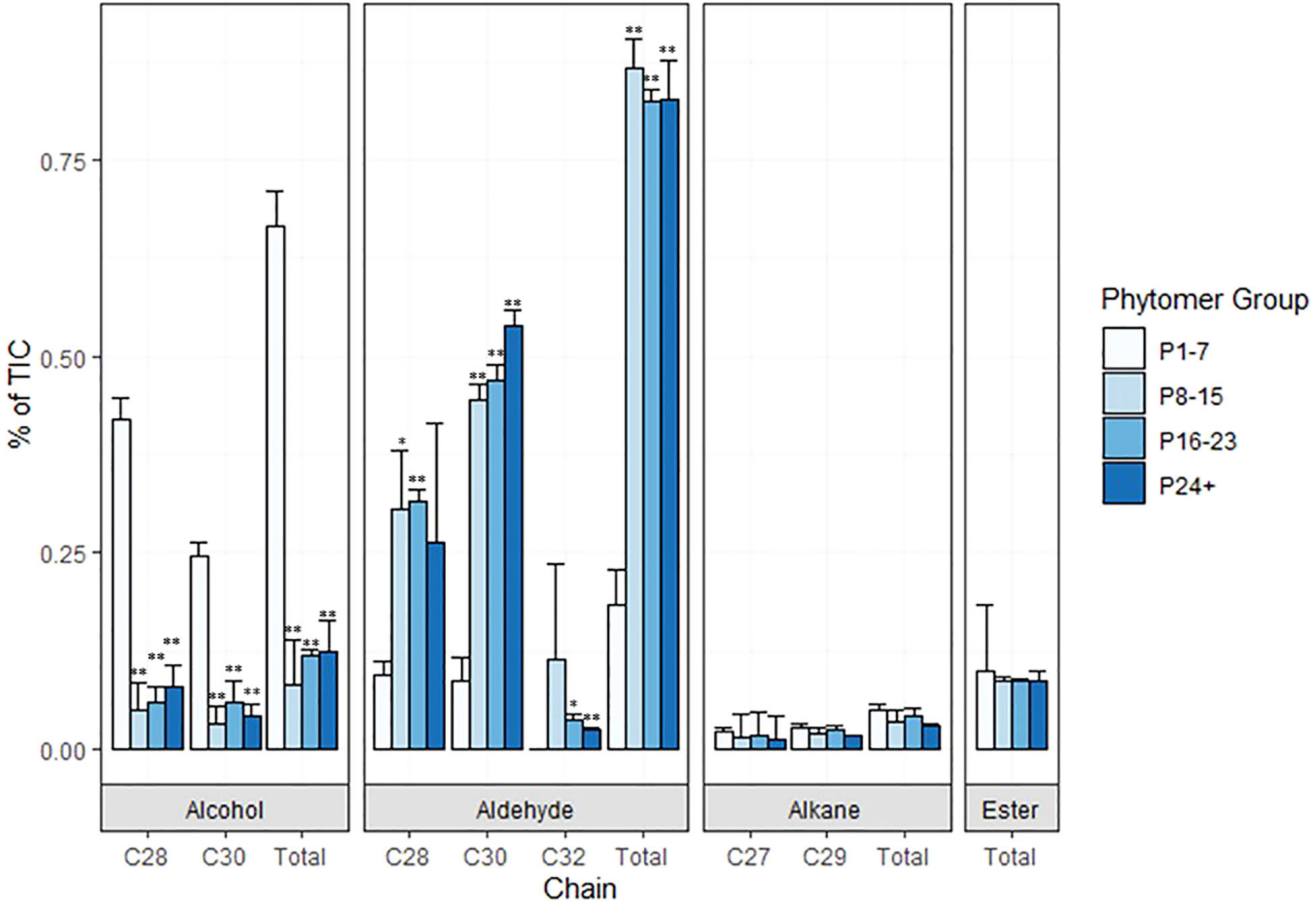
Composition of sorghum stem wax on phytomers of TX08001 120 DAE plants. Wax was extracted from groups of phytomers of increasing age and development (1-7, 8-15, 16-23, and 24+) for composition analysis. The relative abundance of wax components (C28/C30 alcohols, aldehydes, alkanes, esters) extracted from stems of each set of phytomers is represented by bar graphs (P-values designated by asterisks as follows: *<.01, **<.001, P1-7). % of TIC (Total Ion Current) is calculated from area under the peak with local background subtraction.

Preliminary analyses of wax composition on internodes at various stages of development were conducted in 2019/2020. In 2022, TX08001 plants were grown in rhizotrons in a new automated phenotyping greenhouse with high side walls that enabled vegetative growth of tall bioenergy sorghum for 120 days prior to wax composition analysis. Plant shoots were harvested, leaves and leaf sheaths removed from stems and wax was extracted from stem segments associated with P1-7, P8-15, P16-P23 and P24+. GC-MS analysis of wax extracts collected from P1-7 revealed that C28/30 primary alcohols were the most abundant wax component during early stem development (**Figure 4**; **Supplementary Figure 2**). The wax alcohols consisted primarily of octacosanol and melyssil alcohol (C28 and C30 primary alcohols, respectively), with small amounts of octacosanal and triacotonal (**Figure 4**). In contrast, wax from older more developed internodes was enriched in long chain fatty aldehydes (C28, C30) (**Figure 4**). Wax esters and alkanes were relatively minor wax components that did not change significantly in relative abundance during internode development.

### Sorghum wax pathway genes expressed in the stem epidermis

Sorghum homologs of genes involved in stem wax biosynthesis are annotated in the BTx623 reference genome (Phytozome v3.1) (McCormick et al., 2018). To confirm and refine the annotations, sorghum homologs of validated Arabidopsis and maize wax pathway genes were identified and used as the starting point for phylogenetic analysis. Sorghum homologs that contained the same PFAM domains as the validated wax pathway genes and that had BLAST X score e-values <1e^-20^ were included in the analysis. The phylogenetic relationships among the sorghum, Arabidopsis, rice, and maize wax pathway homologs were analyzed using Mega-X (Tamura et al., 2011; Kumar et al., 2018; Tamura et al., 2021) (**Supplementary Figures 3A–Z**). This approach identified sorghum gene homologs encoding long chain acyl-CoA synthetases (*LACS1/2/4/9*), subunits of the fatty acid elongase (*CER6, KCR1, PAS2, ECR/CER10, CER2*), the wax alcohol forming pathway (*CER4, WSD1*), the wax alkane pathway (*CER3, CER1, MAH1, WSD1)* and wax transporters (*CER5/ABCG12, ABCG11, LPTG*) (**Supplementary Table 1**). This method identified Sobic.006G155700 as a wax transporter (*WBC11/ABCG11*) consistent with sorghum mutant analysis (Mizuno et al., 2013).

One goal of this study was to identify wax pathway genes involved in stem epicuticular wax formation. Therefore, the sorghum homologs of genes involved in wax biosynthesis and transport were analyzed to identify gene family members that were differentially expressed in stem epidermal cells. This was accomplished by comparing the expression of each wax pathway gene family member in sorghum stem internode epidermal cells (Ep) to non-epidermal cell types [i.e., pith parenchyma (PP), xylem fibers (XF), xylem/vascular parenchyma (XP) and the phloem (Ph)] using data derived from LCM-based RNAseq analysis (Fu et al., 2023). The subset of the sorghum wax pathway gene homologs in each gene family that were differentially expressed in stem epidermal cells was identified and targeted for further analysis (**Supplementary Table 2**). The sorghum triterpene synthase gene (Sobic.008G142400) that produces cuticular triterpenoids (Busta et al., 2021) and the sorghum gene (Sobic.001G228100) encoding a GDSL-lipase required for wax accumulation on the leaf sheath (Jiao et al., 2017) were also differentially expressed in epidermal cells (**Supplementary Table 2**).

### Wax pathway gene expression during stem development

Expression of the sorghum wax pathway genes during stem development was characterized using RNAseq data derived from developing vegetative phase stems of R07020 (Casto et al., 2018). R07020 stem tissue was collected from two apical internode sections (Int#1, Int#2) that had not yet initiated rapid internode elongation, apical to basal sections from Int#3 (i.e., 3-1, 3-2, 3-3, 3-4, 3-5), an internode that was undergoing rapid elongation, and apical to basal sections from Int#4, an internode that was fully elongated (Casto et al., 2018). *SbLACS1* was expressed at ~16 TPM in Int#1/2 and at somewhat higher levels in most sections of Int#3 and Int#4 (~29-53 TPM) (**Supplementary Table 3**). *SbLACS4* expression was also relatively high in Int#1-4 (19-87 TPM). In contrast, *SbLACS2* and *SbLACS9* were expressed at 5-21 TPM in Int#1-3, then at lower levels in the upper older sections of Int#4 (1-5 TPM). *SbPAS2* and *SbECR* were expressed at relatively high levels in Int#1-4 (53-272 TPM, 134-273 TPM respectively) (**Supplementary Table 3**).

**Figure 5:**
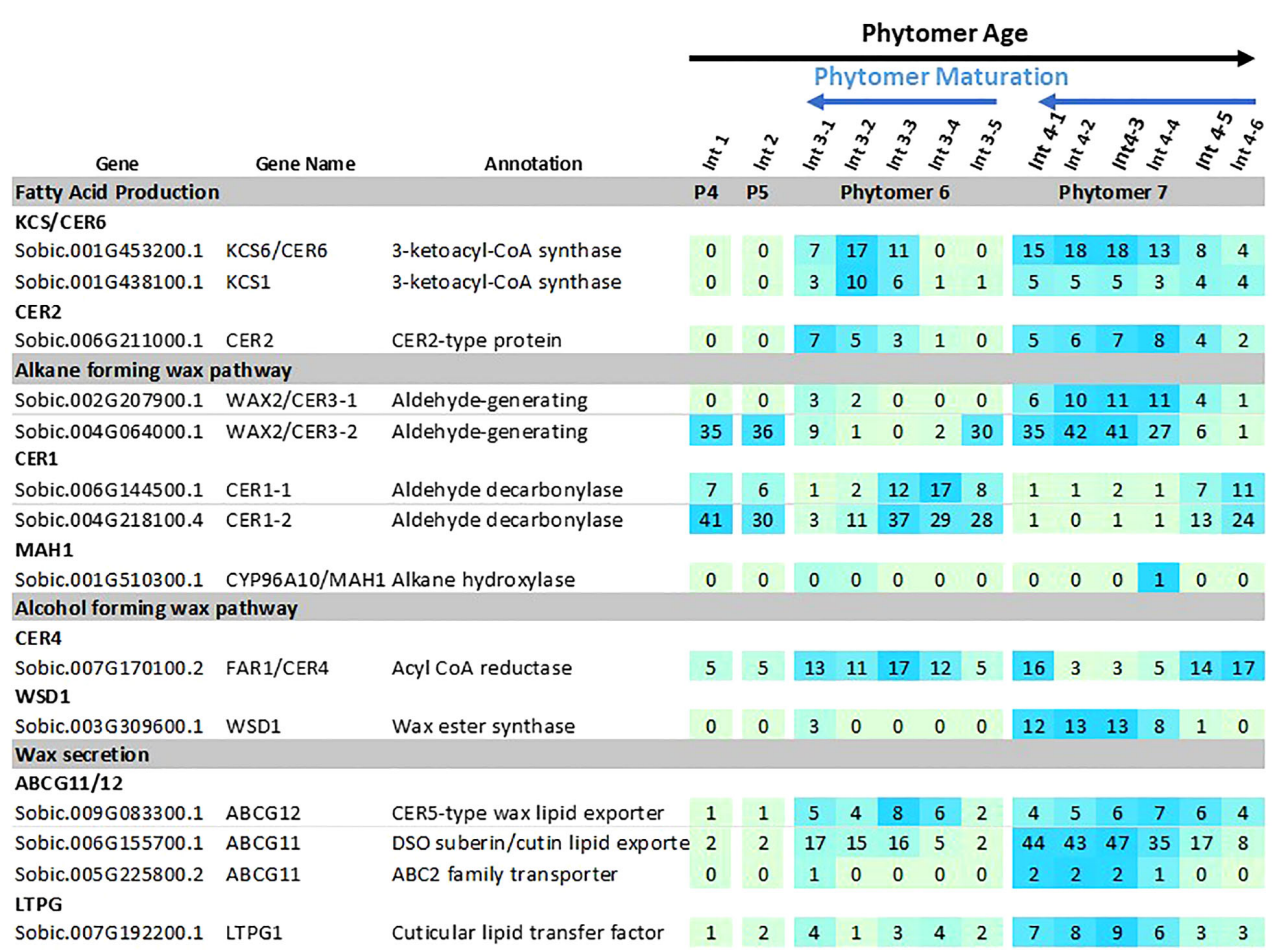
Expression of sorghum wax pathway genes during stem internode development of R07020. Expression (TPM) of wax pathway genes in R07020 stem internodes 1-4 (Casto et al., 2018). Stem internodes 3 and 4 were divided into sections where Int#3-1 and Int#4-1 are from the upper end of the internode and Int3-5 and Int4-6 were from the lower end of the internode, a region spanning the growing zone. Cells are shaded in light green>blue (low to high) scale as relative expression within the data set. Additional information on wax pathway gene expression is found in **Supplementary Tables 1**–**3**).

Several genes in the wax pathway showed more extensive variation in expression during stem internode development (**Figure 5**). For example, expression of *SbKCS1*, *SbKCS6* and *SbCER2* was <1 TPM in Int#1/2, then expression increased in the upper more fully developed sections of Int#3 and Int#4. *SbKCR1* was expressed at ~2 TPM in Int#1/2 and at 4-fold higher levels in the upper more fully developed portion of Int#4. *SbCER3-1, SbWSD1*, *SbABCG12, SbABC11* had a similar developmental pattern of expression with low expression in Int#1/2 and 5-20-fold higher expression in the fully elongated, older, more developed portions of Int#3/4. Other genes in the alkane and alcohol wax pathways had more complex patterns of expression. For example, *SbCER3-2* was highly expressed in Int#1/2 and the basal section of Int#3 that spans the intercalary meristem, but also in the upper more fully developed portion of Int#4 where *SbCER3-1* is also highly expressed in conjunction with *SbCER1-2*. In contrast, *SbCER1-1* and *SbCER1-2* were expressed at high levels in Int#1/2 and the basal growing zone sections of Int#3 and Int#4 but at lower levels in the upper more developed portions of Int#3/4. *SbCER4* was expressed in Int#1/2, throughout most of Int#3, then at the base and top of Int#4 (stem node tissue).

### Gene regulatory network analysis

**Figure 6:**
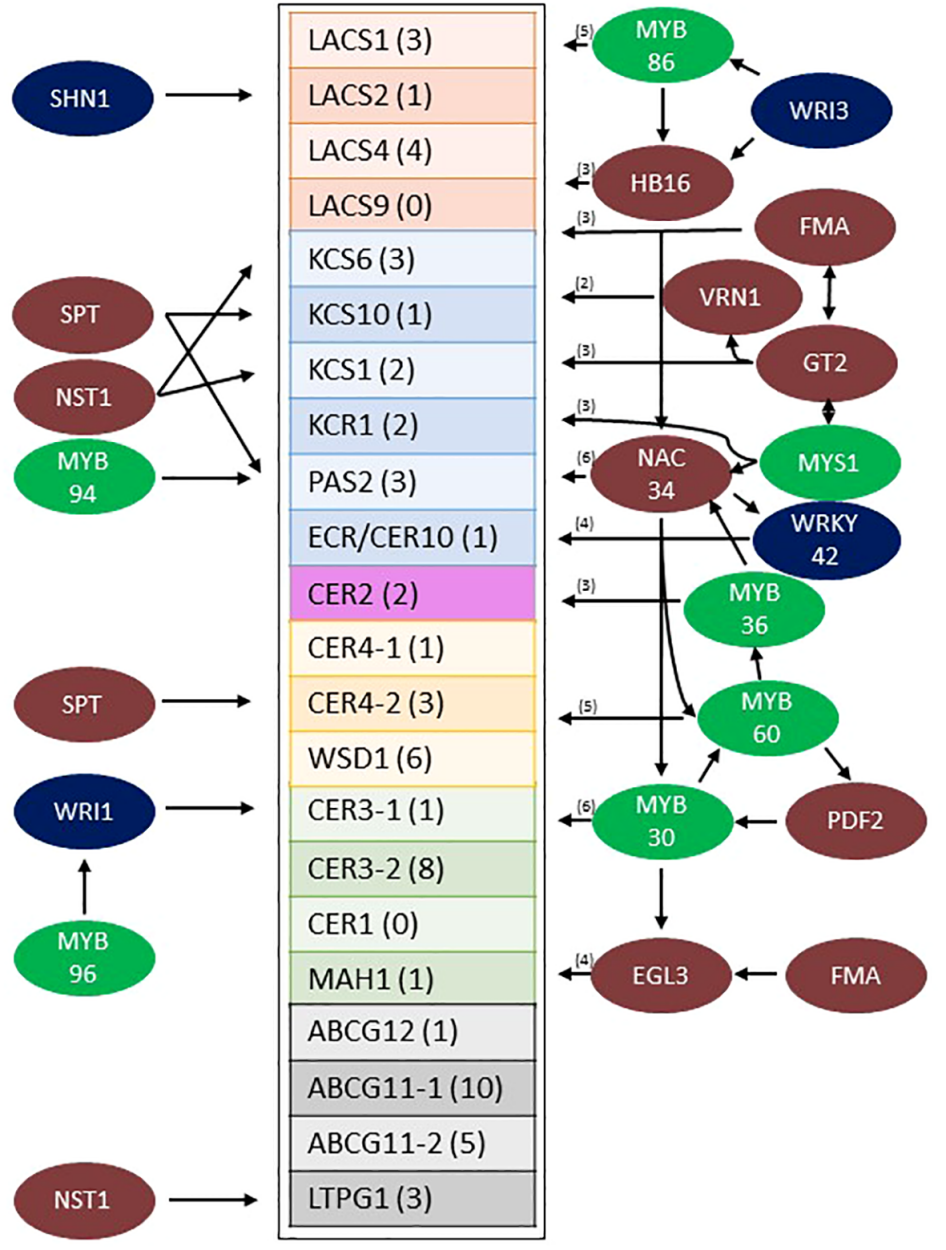
Model of the predicted sorghum stem wax pathway gene regulatory network from Wray LCM data (Fu et al., 2023). Sorghum wax pathway genes that are differentially expressed at higher levels in the stem epidermis are boxed (the number of TF connections to each gene is shown in parentheses). TFs (colored circles, left side) are shown with arrows indicating connections to specific wax pathway genes. TFs with 2-6 predicted connections to wax pathway genes are shown on the right (the number of connections in parentheses). Specific connections between TFs shown to the right and their target wax pathway genes are shown in **Supplementary Figures 5A–D**. MYB factors are color coded green, previously identified EW associated TFs are colored blue and other TFs are colored brown. EW, Epicuticular Wax; TF, Transcription Factor.

Transcription factors expressed in the stem epidermis that potentially regulate wax pathway gene expression were identified by gene regulatory network analysis (Varala et al., 2018; Arachchilage et al., 2020; Yu et al., 2022). Sorghum stem cell type transcriptome data (Casto et al., 2018) was used to determine that approximately 1,669 genes were differentially expressed at >5-fold higher levels in the stem epidermis compared to other stem cell types (cutoffs = 5 transcripts per million [TPM], 0.05 false discovery rate [FDR]). After applying a Pearson Correlation Coefficient (PCC) threshold of > 0.9, 626 genes with 1416 edges were retained in the network. Sorghum wax pathway genes and genes encoding many transcription factors (TFs) were differentially expressed in sorghum stem epidermal cells. Potential connections between the TFs and wax pathway gene promoters were identified through gene regulatory network (GRN) analysis (**Supplementary Figure 4**). The sorghum stem epidermis wax pathway GRN included homologs of TFs that have previously been identified as regulators of wax biosynthesis in Arabidopsis (i.e., MYB96/94, WRI1, SHN1, WIN1) (Broun et al., 2004; Kannangara et al., 2007; Seo et al., 2011; Lee and Suh, 2015; Lee et al., 2016; Park et al., 2016). Connections between these TFs and their target genes in the stem wax pathway are shown in **Figure 6** (left side). Other TFs (i.e., WRI3, MYS1, MYB30) that are involved directly or indirectly in wax biosynthesis (Raffaele et al., 2008; Park et al., 2016; Liu et al., 2022) were part of more complex sub-networks that had 47 predicted connections to genes in the wax pathway (**Figure 6**, right). Four sub-networks were tentatively identified that included specific subsets of TFs; (A) WRI3/MYB86/HB16, (B) MYS1/G2-GARP/FMA/VRN1, (C) NAC034, which is potentially regulated by HB16/MYS1/MYB36 and connected to genes encoding MYB30/MYB60/WRKY42, and (D) MYB30/MYB60/EGL3, with predicted regulation by PDF2, NAC034, and FMA. The network of TFs was connected to every gene in the wax pathway except *CER1* and *LACS9*. Most wax pathway genes were regulated by several TFs and *CER3-2* (8 connections) and *ABCB11-1* (10 connections) were most highly connected to the TF regulatory network. The specific wax pathway genes that could potentially be regulated through each of the four subnetworks were identified (**Supplementary Figures 5A–4D**). Since MYB-factors play a central role in the sorghum stem wax GRN (MYB30/32/36/60/86/94/96/MYS1) the phylogenetic relationships among the sorghum proteins and Arabidopsis MYB proteins were analyzed using MEGA-X (**Supplementary Figure 6**) (Singh et al., 2020). This analysis showed that sorghum and Arabidopsis MYB30/60/94/96 clustered in the same clade whereas SbMYB32, SbMYB36, and SbMYB86 clustered with their Arabidopsis homologs in other clades.

## Discussion

Most prior studies of sorghum wax focused on grain sorghum to better understand the contribution of leaf wax to sorghum’s drought tolerance. Early studies correlated higher leaf blade wax load to reduced non-stomatal transpiration from leaf surfaces (Nobel and Jordan, 1983; Jordan et al., 1984). Sorghum leaf sheath wax has also been characterized in part because the external surface of the sheath has a heavy wax load and diverse wax morphologies (Jenks et al., 1994; Jenks et al., 2000). Grain wax has also been analyzed to determine its potential as a co-product of grain production (Hwang et al., 2002; Xiao et al., 2020). However, sorghum stem wax has been studied to a limited extent most likely because dwarf grain sorghum genotypes have short stems that are covered by leaf sheaths. In contrast, bioenergy sorghum hybrids developed for biomass/bioenergy production have 4-5 m long stems (x 20-30 mm diameter) that account for ~25% of the above ground plant surface area. Stems of bioenergy sorghum plants grow continuously by the sequential addition of >40 apical phytomers and internodes during a long growing season (150-200 days). Following canopy closure, leaf blades and leaf sheaths lower in the canopy senesce exposing wax covered stems to the environment. Exposed stem surfaces accumulate a thick wax plate that often covers internode stomata potentially reducing water loss from internode surfaces and contributing to biotic resistance by blocking stomatal pores. Given the potential importance of stem wax to biotic/abiotic resilience and its utility as a valuable co-product, the current study was undertaken to characterize sorghum stem wax and the regulation of stem wax pathway gene expression.

### Sorghum stem wax load

In Arabidopsis, wax loads on stems (13-32 ug/cm^2^) are >10-fold higher than on leaf blades (0.7-1.5 ug/cm^2^) (Suh et al., 2005; Lee and Suh, 2015). In sorghum, leaf blade wax loads have been reported to vary from 2-25 ug/cm^2^ depending on genotype and stage of development (Nobel and Jordan, 1983; McWhorter et al., 1990; Burow et al., 2008; Burow et al., 2009; Xiao et al., 2020; Busta et al., 2021; Tang et al., 2022). Higher wax loads are reported for sorghum leaf sheaths (~36-206 ug/cm^2^) (McWhorter et al., 1990; Burow et al., 2008; Burow et al., 2009; Xiao et al., 2020). In the current study, wax loads on the leaf sheath associated with internode #8 of the bioenergy sorghum hybrid TX08001 ranged from 41-51 ug/cm^2^ between 60-90 DAE. Stems of TX08001 had a wax load of ~100 ug/cm^2^ at 60 DAE (internode #8), ~197 ug/cm^2^ at 90 DAE (internode #8) and ~215 ug/cm^2^ at 120 DAE (internode #23). Therefore, sorghum wax loads are much higher than Arabidopsis, but like Arabidopsis, sorghum stem wax loads are higher than leaf wax loads. Stems are not thought to be large contributors to non-stomatal transpiration because they are typically shaded by leaf blades and often covered by leaf sheaths that have heavy wax loads. This suggests that stem wax may play an important role in biotic stress resistance in addition to reducing water loss from cuticular surfaces. Of the total shoot surface area at bioenergy sorghum harvest, stem surface area accounts for ~24%, leaf sheaths ~30%, and leaf blades ~46%. High wax loads on bioenergy sorghum stem and leaf sheaths could potentially be extracted at biorefineries to provide high value wax co-products (Cominelli et al., 2008). Recovery of wax from harvested bioenergy sorghum stems, leaf sheaths and leaf blades could allow recovery of 100-200 kg of wax per hectare when the crop is planted at a standard planting density of 132,000 plants/ha (Olson et al., 2012). Sugarcane, a related C4 grass, was estimated to accumulate 40-240 kg of wax per hectare assuming average crop yields (Jetter and Kunst, 2008). Sugarcane stem wax contains higher levels of short chain wax components relative to bioenergy sorghum (Inarkar and Lele, 2012).

### Sorghum stem wax composition

The current study found that sorghum stem wax on younger more apical internodes (P1-7, Int#1-4) contained high levels of C28/30 primary alcohols (~65%) and lower levels of C28/30 aldehydes (~18%) and esters (~7%). In contrast, stem wax on older sorghum internodes (P8+) was highly enriched in C28/30 aldehydes (~80%) and lower amounts of primary alcohols (6-12%) and esters (~8%). Stem wax on sorghum grain genotypes was also reported to be enriched in C28/30 aldehydes (Xiao et al., 2020). The functional significance of accumulation of C28/30 wax aldehydes during stem development is unknown, however, variation in wax composition during organ development is common. For example, the composition of sorghum leaf wax changes during the juvenile/adult phase transition (Avato et al., 1984; Busta et al., 2021). Sorghum juvenile leaf wax is enriched in C28/C30 primary alcohols (80%) whereas wax on adult phase leaves is a mixture of fatty acids, primary alcohols, aldehydes, alkanes, and triterpenoids (Busta et al., 2021). In maize, an increase in the ratio of wax esters/alkanes during leaf development coincided with changes in the water barrier properties of the cuticle (Bourgault et al., 2020). Variation in wax composition during development, on different organ surfaces and species is extensive (Lee and Suh, 2015). Changes in wax chemistry during organ development and variation among organs and plant species could result in differences in wax morphology (Chambers et al., 1975) and the functional properties of wax. As in *Zea mays* (Bourgault et al., 2020), we observed a shift from alkanes to primary alcohols and then aldehydes during stem internode epidermal cell development. The variation in wax composition during development were correlated with changes in the morphology of the SEM visualized epicuticular surface from a smooth wax surface, followed by the appearance of wax tubules on the surface, and finally to a surface with tubules and wax plates.

### Identification of sorghum stem wax pathway genes

A developmental study of wax accumulation and gene expression in the Arabidopsis stem epidermis was highly successful in identifying genes involved in epicuticular wax biosynthesis (Suh et al., 2005). Therefore, in the current study a similar approach was used to identify sorghum wax pathway genes that contribute to stem wax accumulation. Initially, sorghum homologs of known wax pathway genes were identified using BLAST X, PFAM domain information, and phylogenetic analysis. This approach identified small sorghum gene families for nearly every step in the wax pathway. Wax pathway gene family members that were differentially expressed in stem internode epidermal cells were identified using transcriptome profiles of sorghum stem internode cell types (Fu et al., 2023). Many of the sorghum wax pathway genes identified using this method were homologs of Arabidopsis genes expressed in the stem epidermis (Suh et al., 2005). For example, in Arabidopsis, *AtLACS1, AtLACS2* and *AtLACS3* were expressed in the stem epidermis. In sorghum, homologs of *AtLACS1, AtLACS2, AtLACS4* and *AtLACS9* were expressed in the stem epidermis where they could contribute to cutin and wax formation. AtLACS1 and AtLACS2 synthesize long-chain acyl-CoAs for wax and cutin biosynthesis (Lu et al., 2009; Weng et al., 2010) and AtLACS1 and AtLACS9 contribute to the synthesis of tryphine lipids in the pollen coat (Jessen et al., 2011). *SbKCS1, SbKCS6* and *SbKCS10* are also differentially expressed in the stem epidermis as are the corresponding Arabidopsis homologs (Suh et al., 2005). KCS is considered the rate limiting enzyme in the FAE complex and KCS gene family members influence the type of VLCFAs that are produced (Guo et al., 2016). KCS1, for example, primarily catalyzes the elongation of C20-C26 VLCFAs, whereas KCS6, encoded by the *KCS*-gene with the highest expression in the sorghum stem epidermis, generates VLCFAs longer than C24, consistent with its role in wax biosynthesis (Trenkamp et al., 2004; Huang et al., 2022). *SbKCS10* is a homolog of *FIDDLEHEAD*, a gene that impacts the development of the adaxial epidermis of rosette leaves and trichomes (Yephremov et al., 1999). Sorghum genes encoding the other subunits of the FAE (*SbKCR1*, *SbPAS2, SbECR*) and *SbCER2*, a gene that increases the production of VLCFA with chain lengths greater than C28 (Tacke et al., 1995; Pascal et al., 2013; Haslam et al., 2015; Xue et al., 2017; Alexander et al., 2020), were also differentially expressed at higher levels in the stem epidermis compared to non-epidermal cells. In addition, another *SbKCR1* homolog (Sobic.004G203900) was highly expressed throughout the stem in Int#1-Int#4.

In the wax alkane pathway, CER3 generates aldehydes, CER3:CER1 complexes convert aldehydes to alkanes, and MAH1 converts wax alkanes to secondary alcohols and ketones (Bernard and Joubes, 2013). In Arabidopsis, *AtCER3* and *AtCER1* were expressed at similar levels in the stem epidermis consistent with accumulation of high levels of C29 wax alkanes (32%) relative to C28/30 aldehydes (5%) (Lee and Suh, 2015). In sorghum two *SbCER3* and two *SbCER1* homologs were differentially expressed in the sorghum stem epidermis. However, unlike Arabidopsis, *SbCER3* expression was 58-fold higher than *SbCER1* consistent with elevated accumulation of C28/30 aldehydes in the sorghum stem epidermis relative to wax alkanes. Sorghum stem wax also accumulated only low levels of wax secondary alcohols and ketones consistent with very low expression of the sorghum homolog of *AtMAH1*, a gene involved in generating secondary wax alcohols and ketones (Greer et al., 2007; Wen and Jetter, 2009). The formation of wax primary alcohols is mediated by an NADH-dependent fatty acyl-coA reductase (FAR) (Kolattukudy, 1971). In Arabidopsis AtCER4/FAR3 mediates wax primary alcohol formation whereas in Triticeae, *TaFAR1* encodes the enzyme that mediates production of primary alcohols in the cuticle (Wang et al., 2015). The sorghum homolog (Sobic.005G063300) of *AtCER4/FAR3* was expressed at very low levels in the sorghum stem epidermis. In contrast, the sorghum homolog (Sobic.007G170100) of *TaFAR1* (Wang et al., 2015) was differentially expressed in the epidermis of sorghum stems consistent with a role in stem wax primary alcohol formation. A sorghum homolog (Sobic.003G309600) of *AtWSD1* (Li et al., 2008), *ZmWSD1, OsWSD1* and *ShWSD1* (Kawahara et al., 2013; Garsmeur et al., 2018; Liu et al., 2021; Li et al., 2022) was differentially expressed in the stem epidermis. Another *SbWSD1* homolog (Sobic.009G226600) that was most closely related to the grass *WSD1* genes was not differentially expressed in stem epidermal cells. The homolog discovery approach employed also led to the identification of sorghum homologs of *ABCG12/11* cutin/wax transporters and *LTPG* that are differentially expressed at higher levels in the stem epidermis.

### Expression of the sorghum stem wax pathway during development

SEM analysis of stem surfaces showed minimal wax accumulation on Int#1/2, young non-elongated stem segments located just below the stem apex. SEM analysis first detected wax accumulation on the upper portion of Int#3, a region of the internode that contains fully elongated cells, but not on the lower portion of internode 3 that spans the intercalary meristem and zone of elongation (Kebrom et al., 2017; Yu et al., 2022). A similar distribution of wax accumulation was observed on internode #4, although larger wax deposits were present on the upper portion of this internode, and wax was visible on a large portion of the internode. Wax on Int#6 and Int#25 was more profuse and wax tubules completely covered the surface of these internodes. Expression of *SbKCS6, SbKSC1, SbCER3-1, SbWSD1* and *SbABCG12/11-1* was low in Int#1/2 followed by increased expression in the upper portion of Int#3 and Int#4, a pattern of wax pathway gene expression correlated with the appearance of wax on the surface of internodes. In contrast, *SbCER1-1* and *SbCER1-2* were expressed at higher levels in Int#1 and Int#2 and the lower portion (growing zone) of Int#3 and Int#4 during development. *SbCER3-2* also showed relatively high expression in Int#1, Int#2, and the lower portion of Int#3. This suggests that *SbCER3-2* and *SbCER1-1/1-2* could generate alkanes that accumulate on Int#1/2 prior to the onset of internode elongation and in the growing zone of Int#3/4. Preliminary MS data showing the presence of alkanes on Int#1 and Int#2 supports this hypothesis and Int#4 during development (**Supplementary Figure 8**). *SbCER3-2* also showed relatively high expression in Int#1, Int#2, and the lower portion of Int#3. There was no visible epicuticular wax accumulation on these nascent internodes suggesting alkane may be a component of intracuticular wax. *SbCER4* was expressed in Int#2/3, then increased in Int#3 followed by decreased expression in Int#4 but with retention of expression in nodal tissues (pulvinus, nodal plexus). *SbCER4* expression in Int#1-3 is consistent with accumulation of primary alcohols in wax early in internode development. The induction of *SbCER3-2* expression (but not *SbCER1*) in the upper portion of Int#4 could contribute to the generation of C28/30 wax aldehydes that become the predominant component of sorghum stem wax on fully developed stem internodes. Taken together, the timing of elevated expression of *SbCER4* before high levels of *SbCER3-2* during internode development is consistent with early accumulation of wax alcohols followed by wax aldehydes (**Figure 4**; **Supplementary Figure #**). Assessment of the impact of continued expression of *SbCER1* and *SbCER4* expression in stem nodes will require targeted analysis of wax composition on this stem tissue to determine if wax alkanes and primary alcohols accumulate preferentially on stem nodal tissues.

### Identification of sorghum transcription factors that regulate stem wax pathway gene expression

Several TFs that regulate wax biosynthesis have been identified in Arabidopsis (i.e., SHN1/WIN1, WRI4, MYB30/94/96, MYS1) (Aharoni et al., 2004; Kannangara et al., 2007; Bernard and Joubes, 2013; Liu et al., 2022). Sorghum homologs of known wax pathway TFs were identified through phylogenetic analysis and then screened to identify gene family members with high expression in the stem epidermis. This approach identified sorghum homologs of several TFs that are known to regulate cuticle/epidermal cell development (i.e., *SbPDF2, SbHDG1, SbEGL3*) (Bernhardt et al., 2003; Nakamura et al., 2006; Singh et al., 2020; Nagata and Abe, 2023) and/or biosynthesis of epicuticular wax (i.e., *SbSHN1.WIN1*, *SbMYB30/94/96, SbWRI1, SbWRI3, SbMYS1).* Gene regulatory network analysis of genes co-expressed in wax producing stem epidermal cells identified predicted TF binding sites in the promoters of genes in the sorghum stem wax pathway as well as among TFs in the network. For example, *SbSHN1/WIN1* was differentially expressed in stem epidermal cells and GRN analysis predicted SbSHN1.WIN1 could regulate the expression of *SbLACS2*, consistent with studies showing AtWIN1 regulates the expression of *AtLACS2* (Kannangara et al., 2007). A different member of the sorghum SHN/WIN gene family, *SbWINL* is highly expressed in leaves, and when over-expressed in Arabidopsis wax crystals accumulated (Bao et al., 2016). Arabidopsis WRINKLED (WRI) TFs regulate tissue specific fatty acid biosynthesis (To et al., 2012) and AtWRI4 was found to activate wax biosynthesis in stems (Park et al., 2016). In the current study, *SbWRI1* and *SbWRI3* were differentially expressed in the stem epidermis and predicted to activate *CER3* (Sobic.002G207900) and *SbMYB86/SbHB16*, TFs that have predicted connections to eight genes in the stem wax pathway. In Arabidopsis, AtMYB86 is involved in secondary cell wall formation (Taylor-Teeples et al., 2015); in tea plants, light regulated flavonoid synthesis (Ye et al., 2021); and in black cottonwood with xylem formation (Wessels et al., 2019). SbMYB86 was not identified in the sorghum stem secondary cell wall gene regulatory network (Fu et al., 2023), however, a role in coordinating wax and flavonoid synthesis in sorghum stem epidermal cells is possible. *SbMYB86* was also connected to *SbGlossy6* (Sobic.008G131299), a gene implicated in wax transport (Li L. et al., 2019). *SbGlossy6* is a homolog of *ZmGlossy6*, a gene that encodes a DUF538 containing protein that influences wax secretion (Li L. et al., 2019). The Arabidopsis *Glossy6* homologs *AtSVB* and *AtSVB2 (ZmGlossy6 homologs)* are modulated by ABA and involved in trichome formation (Hussain et al., 2021).

AtMYB94/96 induce the expression of genes involved in cuticular wax biosynthesis and activate wax accumulation under conditions of water deficit (Koch and Ensikat, 2008; Lee and Suh, 2015; Lee et al., 2016). In the current study, *SbMYB94* and *SbMYB96* were differentially expressed in the sorghum stem epidermis and network analysis indicated these TFs could regulate *PAS2* and *WRI1* expression. Further studies will be needed to determine if SbMYB94/96 play a role in increasing stem wax accumulation in response to drought stress. Phylogenetic analysis showed that *SbMYB30* is closely related to *SbMYB94/96*. *SbMYB30* was differentially expressed in the sorghum stem epidermis and GRN analysis predicted interactions with the promoters of six genes in the wax pathway and *SbGlossy6.* In Arabidopsis, AtMYB30 regulates VLCFA and wax biosynthesis (Raffaele et al., 2008) and also modulates ROS-signaling, PIF-signaling, and hypersensitive responses (Raffaele et al., 2008; Fichman and Mittler, 2020; Yan et al., 2020). GRN analysis predicted *SbMYB30* expression could be regulated by SbPDF2, which could contribute to differential expression in the sorghum stem epidermis. The corresponding *AtPDF2* homolog (AT4G04890) is also differentially expressed in the Arabidopsis stem epidermis (Suh et al., 2005) and is known to help specify shoot epidermal cell differentiation (Ogawa et al., 2015). GRN analysis predicted that SbMYB30 could regulate *SbMYB60* and *SbEGL3* expression and that SbMYB60 could potentially regulate the expression of 5 genes and SbELG3 could potentially regulate the expression of four genes in the wax pathway. In Arabidopsis, *AtMYB60* is expressed in stomatal guard cells (Rusconi et al., 2013) and represses anthocyanin biosynthesis (Park et al., 2008). In sorghum SbMYB60 regulates secondary cell wall formation (Scully ED et al., 2016; Hennet et al., 2020). If *SbMYB60* expression occurs in sorghum epidermal guard cells, the results suggest that SbMYB60 could modulate wax biosynthesis in that cell type in addition to its other functions. *SbEGL3* is a homolog of *AtELG3*, a gene involved in trichome development (Hung et al., 2020), so it is possible that SbEGL3 modulates wax pathway expression in this specialized epidermal cell type. In Arabidopsis leaves, AtMYS1/2 regulates wax production by repressing DEWAX (Liu et al., 2021), a diel regulated gene that represses wax synthesis in conjunction with SPL9 (Li RJ. et al., 2019). *SbMYS1* was expressed in sorghum stem epidermal cells, but *SbDEWAX* expression was not detected. GRN analysis indicated that *SbMYS1* could interact with the promoters of three genes in the epicuticular wax biosynthesis pathway (*SbCER3-2*, *SbABCG11-1/WBC11*, *SbABCG11-2*). *SbMYS1* also was predicted to interact with two TFs, *SbNAC034*, a gene with predicted connections to 6 genes in the wax pathway, and *SbG2-GARP* which encodes a TF with three connections to wax pathway genes. In addition, sorghum homologs of *AtNST1*, a gene involved in secondary cell wall formation/lignin biosynthesis (Scully ED et al., 2016; Hennet et al., 2020) and *AtMYB36*, a gene involved in suberin biosynthesis in root casparian strips in Arabidopsis, were differentially expressed in the sorghum stem epidermis and connected to the wax GRN. Sorghum epidermal cells accumulate secondary cell walls (Fu et al., 2023) and suberin requires synthesis of VLCFA, so there may be regulatory connections between these co-expressed pathways in stem epidermal cells.

The gene regulatory network analysis predictions reported here merit extensive testing using DAPseq, CHIPseq, and other methods to validate the predictions. The analysis could also be refined by collecting transcriptome profiles from specific cell types that comprise the sorghum stem epidermis (i.e., guard cells, silica cells, cork cells, long cells). The current study provides a starting point for an analysis of the regulation of wax pathway expression during sorghum stem development, the basis of sorghum’s heavy wax loads on stems relative to other plant species and among sorghum genotypes, and on different sorghum organ surfaces (i.e., stem wax, adaxial/abaxial leaf and sheath surfaces).

## Data availability statement

The datasets presented in this study can be found in online repositories. The names of the repository/repositories and accession number(s) can be found in the article/**Supplementary Table 6**.

## Author contributions

RC conducted all experiments and analysis. RC and BM contributed to the bioinformatics experiments. SF contributed machine time for initial MS experiments and training. All authors (RC, BM, SF, and JM) contributed to writing the manuscript.

## Supplementary material

Supplementary Figure 1 Sorghum stem development diagram. The youngest phytomers are located immediately below the shoot apex (i.e., Phytomers 1-3, Nascent Leaf) with older and more developed phytomers are located further from the stem apex (i.e., Phytomer 7) (right, phytomer developmental arrow). Internodes associated with each phytomer are comprised of three stem tissues, the nodal plexus, internode and pulvinus. Internodes (Int), the last tissue produced during phytomer development, is formed by the action of an intercalary meristem (IM) (cell division), followed by cell elongation above the IM, and then cell maturation (accumulation of secondary cell walls). The locations where stem samples for SEM analysis (, , ) were taken is shown to the right of the stem diagram. SEM samples shown in were taken from older internodes (Int8, Int25) not shown in **Supplementary Figure 1**.

Supplementary Figure 2 Representative GC-MS spectra used to generate wax composition data that are shown in. **(A)** GC-MS profiles of wax extracted from internodes associated with phytomers 1-7 (P1-7), P8-15, P16-23, and P24+. **(B)** Portion of the GC-MS spectra showing wax esters including Carnauba wax as a reference.

Supplementary Figure 3 Phylogenetic analysis of sorghum wax pathway gene homologs (e-values <-20). **(A)** Bootstrap (BS) Arabidopsis E-Score and BS values for Arabidopsis and *Zea mays*. Values contained in [] is the BS value without added *Zea mays* genes, _†_ delineates predictive *Zea mays* gene in literature by expression, _‡_ delineates predictive gene from *Saccharum officinarum*, () delineates which supplemental figure panel specifies the tree for the gene family. Green highlighted genes designate where single candidates were selected and are green highlighted in the associated tree. **(B)** Phylogenetic tree of *CER6* gene family. **(C)** Phylogenetic tree of *KCR1* gene family. **(D)** Phylogenetic tree of *PAS2* gene family. **(E)** Phylogenetic tree of *CER10* gene family. **(F)** Phylogenetic tree of CER2 gene family. **(G)** Phylogenetic tree of LACS1 gene family. **(H)** Phylogenetic tree of *CER1&3* gene family. **(I)** Phylogenetic tree of *MAH1* gene family. **(J)** Phylogenetic tree of *CER4* gene family. **(K)** Phylogenetic tree of *WSD1* gene family. **(L)** Phylogenetic tree of *WSD1* gene family that includes *Saccharum officinarum.* **(M)** Phylogenetic tree of *ABCG11&12* gene family. **(N)** Phylogenetic tree of *LTPG1* gene family.

Supplementary Figure 4 Cytoscape figure of the wax pathway gene regulatory network (GRN). Transcription factor (blue nodes) connections to target gene promoters are denoted by lines. Selected genes in the wax pathway are noted.

Supplementary Figure 5
**(A-D):** Sub-networks of the wax pathway GRN showing connections between TFs and wax pathway genes. The four networks contain the following TFs: **(A)** WRI3/MYB86/HB16/NAC34, **(B)** GT2/VRN1/EGL3/FMA/MYS1, **(C)** NAC34/WRKY42/MYB30/HB16/MYS1/MYB36/MYB60, **(D)** MYB30/MYB60/EGL3/NAC34/PDF2. All depicted connections meet the threshold of PCC>.9 and FDR<.05.

Supplementary Figure 6 Phylogenetic analysis of sorghum MYB factors. Same color arrows denotes homologous Arabidopsis and Sorghum MYB factors. Arabidopsis MYB factors MYB30, MYB32, MYB36, MYB60, MYB60-2, MYB86, MYB94 and MYB96 were used in the analysis.

Supplementary Figure 7 Unedited SEM (no contrast adjustment).

Supplementary Figure 8 GC-MS of BSTFA derivatized wax extracted from sorghum stems on DB-5MS. **(A)** Internode 2, **(B)** Internode 3, **(C)** Internode 4, **(D)** Internode 25. Red arrows indicate C28/30 wax alcohols and blue arrows point to the derivatized C28/30 aldehydes. Purple arrow indicates Nonacosane.

Supplementary Figure 9 Bargraph of selected genes from **Supplementary Table 3**. **(A)** CER6 (Sobic.001G453200.1) & **(B)** WSD1 (Sobic.003G309600.1) the bargraphs on the left show the TPM as a function of internode number and position. The bargraphs on the right show core vs rind expression of the gene within the Internode #4.

Supplementary Table 1 List of sorghum gene family homologs of wax biosynthetic genes identified in other species. Genes that are differentially expressed in the stem epidermis are highlighted in green. The sorghum to Arabidopsis gene Blast X E-values are listed. The fold change of RNA expression in rind/core of internode 4 of R07020 (red means additive expression, due to no core expression). Gene expression (TPM) of wax pathway genes in stem cell types (epidermis, pith, xylem, vascular bundle parenchyma, phloem, fibers) is shown to the right (based on LCM analysis, see methods) (Casto et al., 2018; Fu et al., 2023).

Supplementary Table 2 List of sorghum gene homologs of wax biosynthetic genes that are differentially expressed in the stem epidermis (Fu et al., 2023).

Supplementary Table 3 Expression of sorghum homologs of wax pathway genes during stem development. Relative gene expression (TPM) in internode 1 (Int1), internode 2 (Int2), sections of internode 3 (3-1 (top) to 3-5 (lower portion of Int3) and internode 4 (4-1 to 4-6). Intensity of blue highlighting indicates relative expression. The transcriptomic data for this analysis was generated in a previous study (Casto et al., 2018).

Supplementary Table 4 GRN analysis identifying first tier connections between transcription factors and the wax biosynthetic genes that are predicted to interact with. Grey boxes delineate wax biosynthetic gene families, a blue box designates a predicted wax biosynthetic gene, with the white boxes are TFs with a PCC of.95 to the biosynthetic gene.

Supplementary Table 5 Table showing predicted interactions between TFs that are part of the wax pathway GRN (listed at the top of the Table) and wax pathway genes (on the left) that the TFs are connected to (blue highlighted boxes indicate a predicted interaction).

Supplementary Table 6 SRA information used in this study. **(A)** R07020 SRA information (Casto et al., 2018). **(B)** LCM SRA information (Fu et al., 2023).
