# Supplementary material for: Epicuticular wax accumulation and regulation of wax pathway gene expression during bioenergy Sorghum stem development
Source: Front Plant Sci. 2023 Oct 23;14:1227859. doi: 10.3389/fpls.2023.1227859 (PMC10626490; doi:10.3389/fpls.2023.1227859)
Supplement: Supplementary Figure 1 — Sorghum stem development diagram. The youngest phytomers are located immediately below the shoot apex (i.e., Phytomers 1-3, Nascent Leaf) with older and more developed phytomers are located further from the stem apex (i.e., Phytomer 7) (right, phytomer developmental arrow). Internodes associated with each phytomer are comprised of three stem tissues, the nodal plexus, internode and pulvinus. Internodes (Int), the last tissue produced during phytomer development, is formed by the action of an intercalary meristem (IM) (cell division), followed by cell elongation above the IM, and then cell maturation (accumulation of secondary cell walls). The locations where stem samples for SEM analysis (, , ) were taken is shown to the right of the stem diagram. SEM samples shown in were taken from older internodes (Int8, Int25) not shown in Supplementary Figure 1 . [file DataSheet_1.zip › Supplementary Figure 5.pptx]

## Slide 1
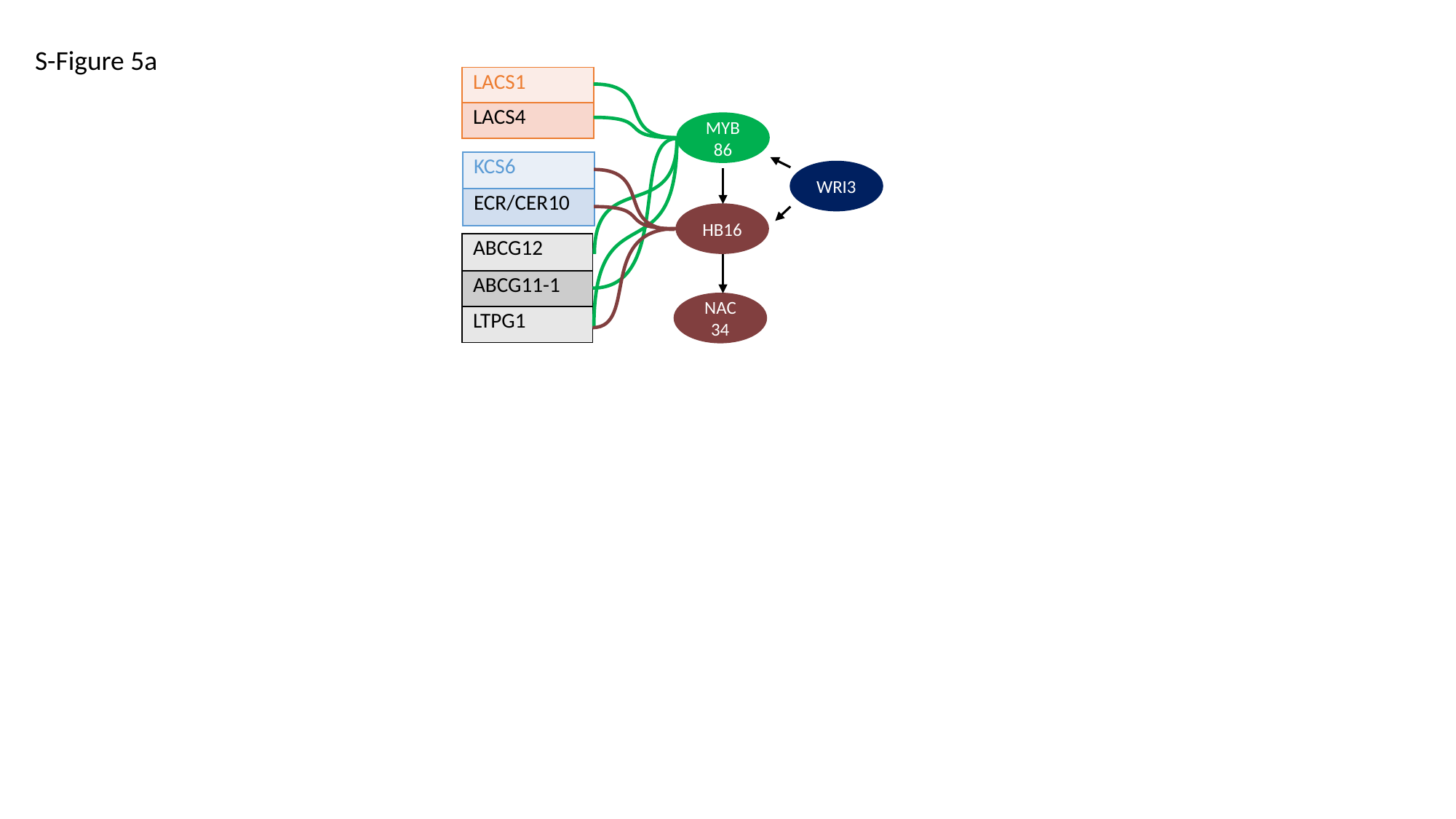

S-Figure 5a
| LACS1 |
| --- |
| LACS4 |
MYB
86
| KCS6 |
| --- |
| ECR/CER10 |
WRI3
HB16
| ABCG12 |
| --- |
| ABCG11-1 |
| LTPG1 |
NAC
34
EGL3
FMA

## Slide 2
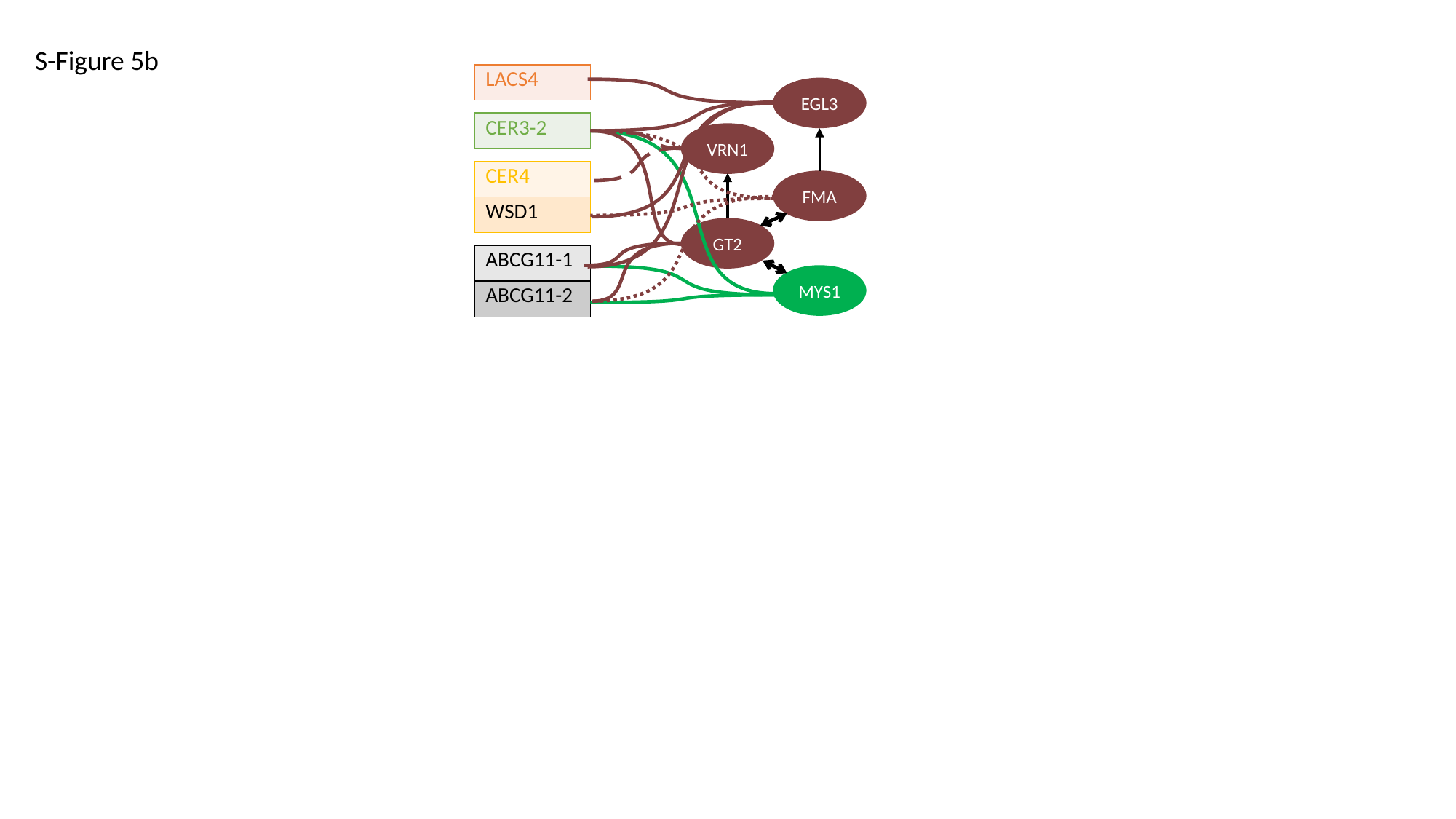

S-Figure 5b
| LACS4 |
| --- |
EGL3
| CER3-2 |
| --- |
VRN1
| CER4 |
| --- |
| WSD1 |
FMA
GT2
| ABCG11-1 |
| --- |
| ABCG11-2 |
MYS1
EGL3
FMA

## Slide 3
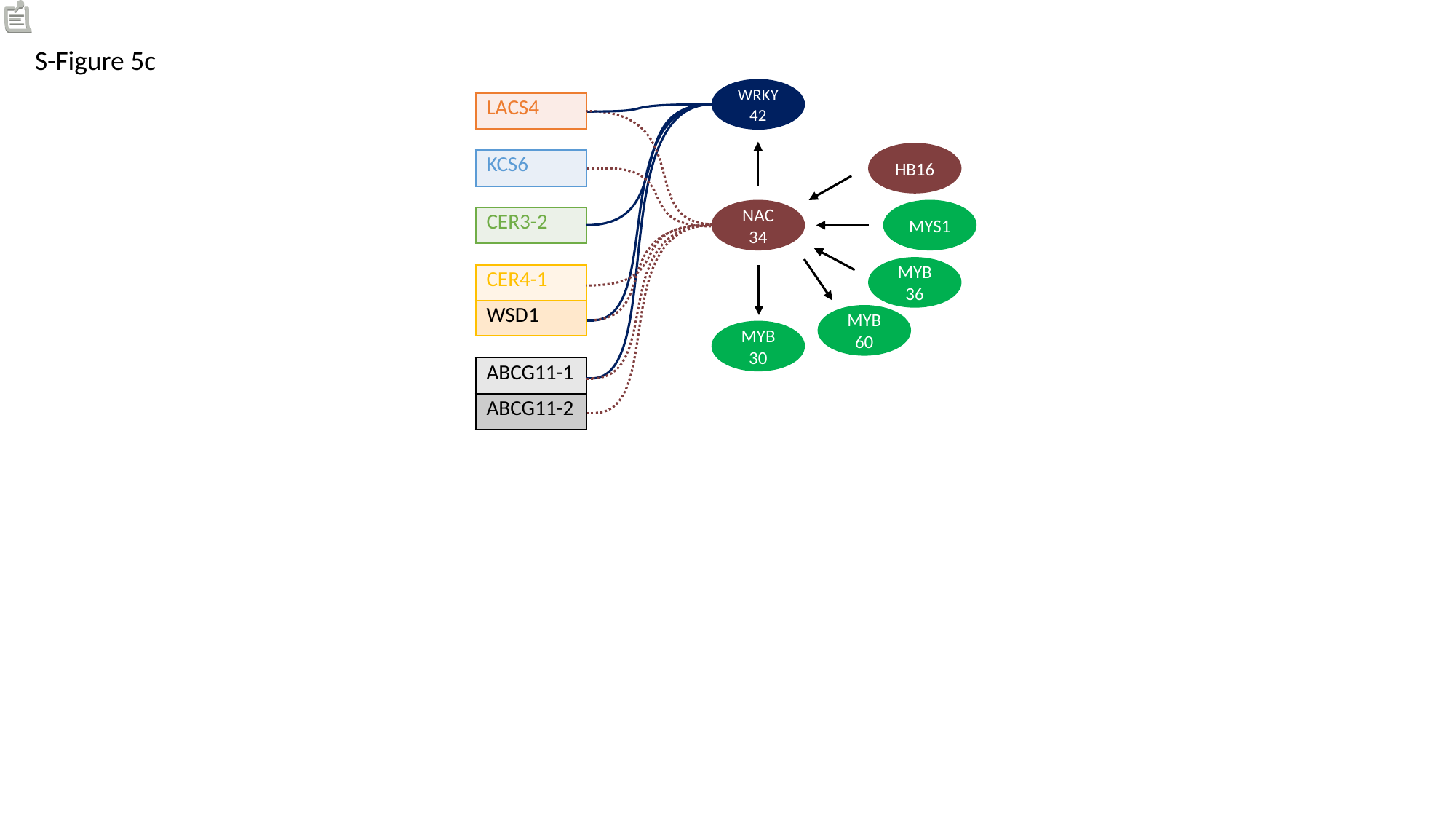

S-Figure 5c
WRKY42
| LACS4 |
| --- |
HB16
| KCS6 |
| --- |
NAC
34
MYS1
| CER3-2 |
| --- |
MYB
36
| CER4-1 |
| --- |
| WSD1 |
MYB
60
MYB
30
| ABCG11-1 |
| --- |
| ABCG11-2 |

## Slide 4
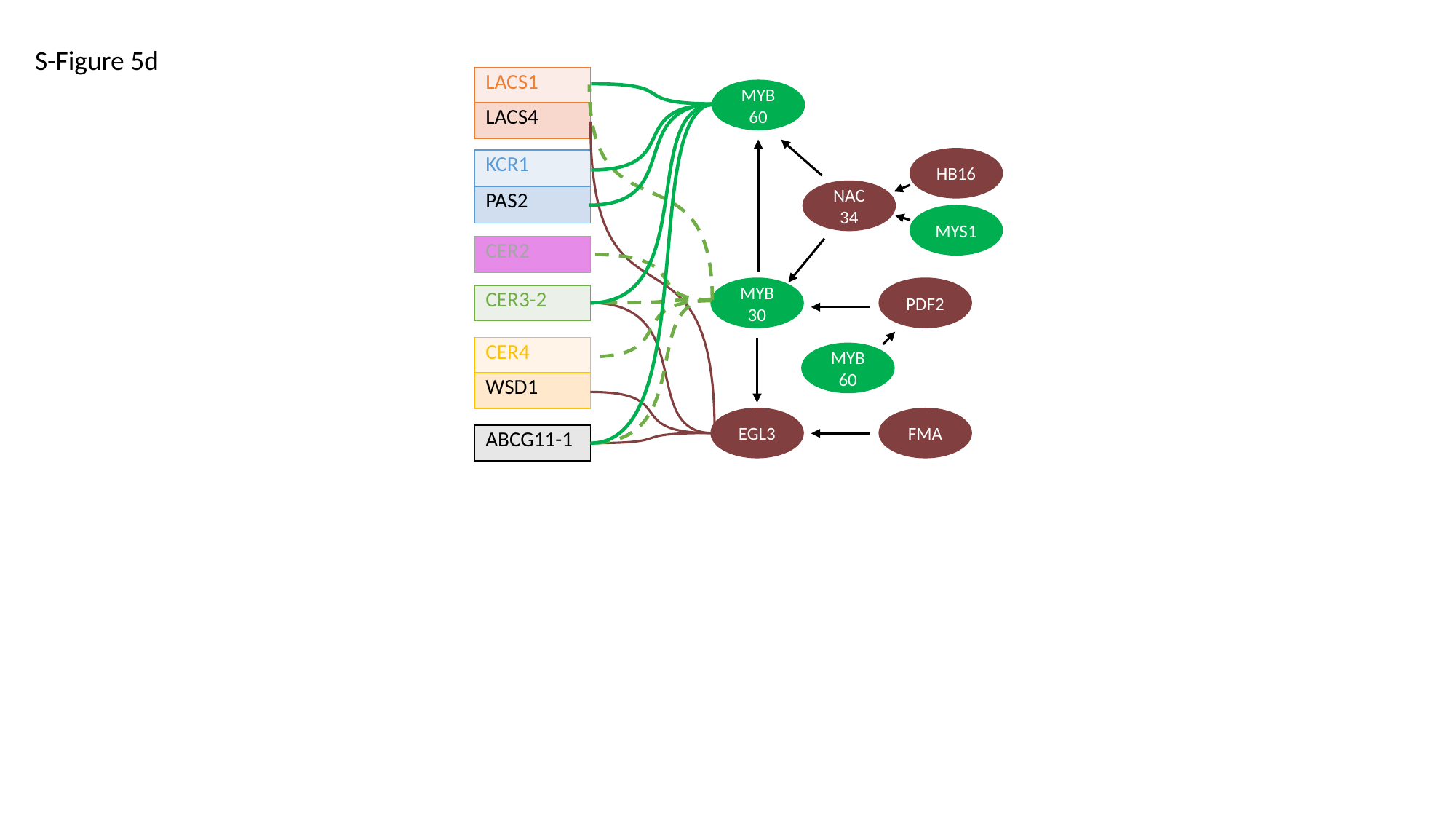

S-Figure 5d
| LACS1 |
| --- |
| LACS4 |
MYB
60
HB16
| KCR1 |
| --- |
| PAS2 |
NAC
34
MYS1
| CER2 |
| --- |
MYB
30
PDF2
| CER3-2 |
| --- |
| CER4 |
| --- |
| WSD1 |
MYB
60
EGL3
FMA
| ABCG11-1 |
| --- |
EGL3
FMA
