# Supplementary material for: Epicuticular wax accumulation and regulation of wax pathway gene expression during bioenergy Sorghum stem development
Source: Front Plant Sci. 2023 Oct 23;14:1227859. doi: 10.3389/fpls.2023.1227859 (PMC10626490; doi:10.3389/fpls.2023.1227859)
Supplement: Supplementary Figure 1 — Sorghum stem development diagram. The youngest phytomers are located immediately below the shoot apex (i.e., Phytomers 1-3, Nascent Leaf) with older and more developed phytomers are located further from the stem apex (i.e., Phytomer 7) (right, phytomer developmental arrow). Internodes associated with each phytomer are comprised of three stem tissues, the nodal plexus, internode and pulvinus. Internodes (Int), the last tissue produced during phytomer development, is formed by the action of an intercalary meristem (IM) (cell division), followed by cell elongation above the IM, and then cell maturation (accumulation of secondary cell walls). The locations where stem samples for SEM analysis (, , ) were taken is shown to the right of the stem diagram. SEM samples shown in were taken from older internodes (Int8, Int25) not shown in Supplementary Figure 1 . [file DataSheet_1.zip › Supplementary Figure 8.pptx]

## Slide 1
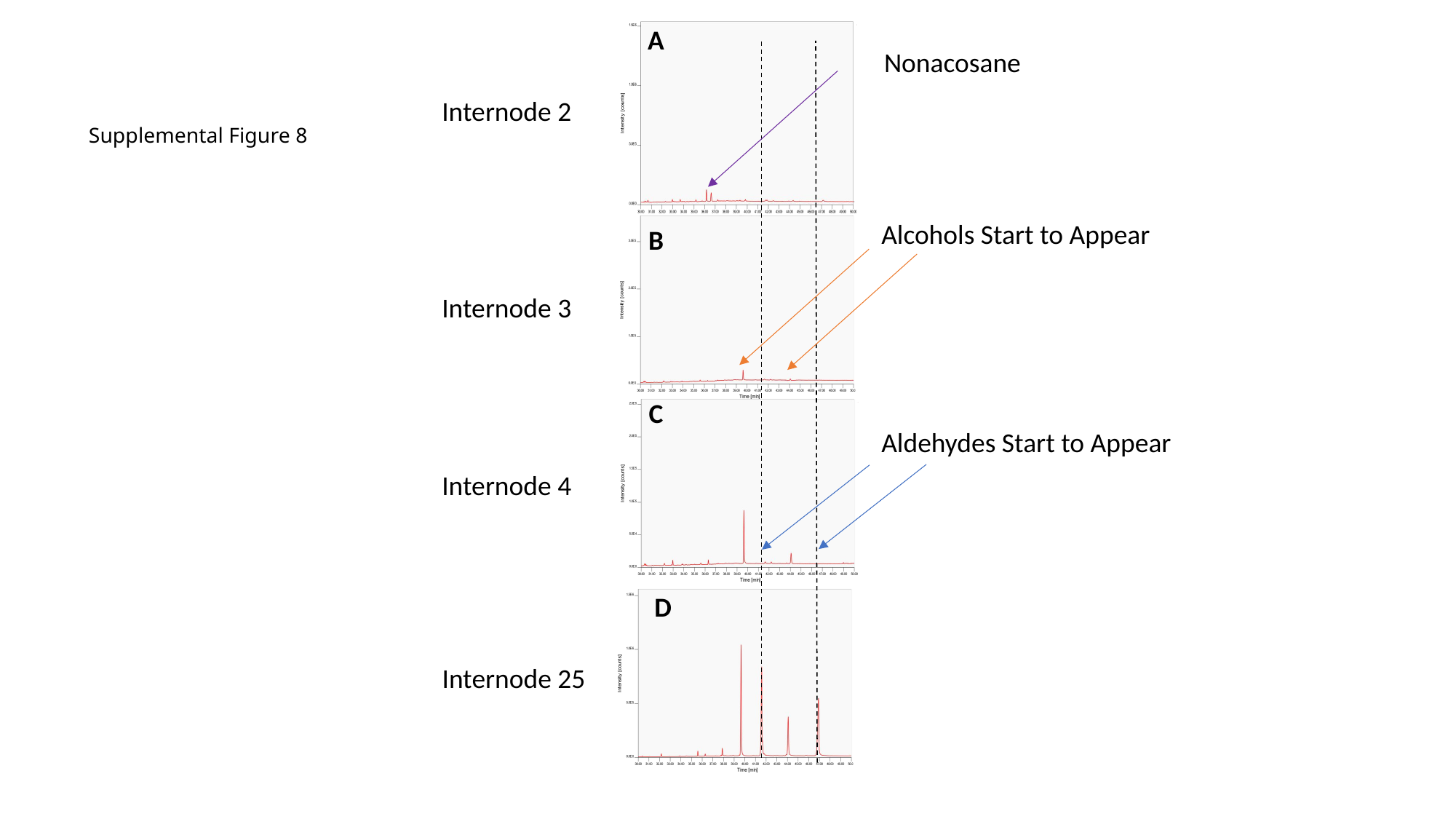

A
Nonacosane
Internode 2
# Supplemental Figure 8
Alcohols Start to Appear
B
Internode 3
C
Aldehydes Start to Appear
Internode 4
D
Internode 25
